# Zr‐MOF Carrier‐Enhanced Dual‐Mode Biosensing Platforms for Rapid and Sensitive Diagnosis of Mpox

**DOI:** 10.1002/advs.202405848

**Published:** 2024-08-09

**Authors:** Huiyi Yang, Judun Zheng, Wei Wang, Jingyan Lin, Jingru Wang, Lunjing Liu, Wenjie Wu, Chengli Zhang, Mingxia Zhang, Yu Fu, Bin Yang, Yuhui Liao

**Affiliations:** ^1^ Molecular Diagnosis and Treatment Center for Infectious Diseases Dermatology Hospital Southern Medical University Guangzhou 510000 China; ^2^ Institute for Engineering Medicine Kunming Medical University Kunming 650500 China; ^3^ National Clinical Research Center for Infectious Disease The Second Affiliated Hospital of Southern University of Science and Technology Shenzhen Third People's Hospital Shenzhen 518000 China; ^4^ NHC Key Laboratory of Metabolic Cardiovascular Diseases Research Ningxia Key Laboratory of Vascular Injury and Repair Research Ningxia Medical University Yinchuan 750004 China

**Keywords:** amplification‐free, colorimetric‐ECL dual‐mode, monkeypox virus, Ru@U6‐Ru/Pt NPs nanotag, signal amplification

## Abstract

Dual‐mode readout platforms with colorimetric and electrochemiluminescence (ECL) signal enhancement are proposed for the ultrasensitive and flexible detection of the monkeypox virus (MPXV) in different scenes. A new nanotag, Ru@U6‐Ru/Pt NPs is constructed for dual‐mode platforms by integrating double‐layered ECL luminophores and the nanozyme using Zr‐MOF (UiO‐66‐NH_2_) as the carrier, which not only generates enhanced ECL and colorimetric signals but also provide greater stability than that of commonly used nanotags. Dual‐mode platforms are used within 15 min from the “sample in” to the “result out” steps, without nucleic acid amplification. The colorimetric mode allows the screening of MPXV with the visual limit of detection (*v*LOD) of 0.1 pM (6 × 10^8^ copies µL^−1^) and the ECL mode supports quantitative detection of MPXV with an LOD as low as 10 aM (6 copies·µL^−1^), resulting in a broad sensing range of 60 to 3 × 10^11^ copies·µL^−1^ (10 orders of magnitude). Validation is conducted using 50 clinical samples, which is 100% concordant to those of quantitative polymerase chain reaction (qPCR), indicating that Ru@U6‐Ru/Pt NPs‐based dual‐mode sensing platforms showed great promise as rapid, sensitive, and accurate tools for diagnosis of the nucleic acid of MPXV and other infectious pathogens.

## Graphical abstract

A new nanotag, Ru@U6‐Ru/Pt NPs is constructed for dual‐mode platforms by integrating double‐layered electrochemiluminescence luminophores and the nanozyme using Zr‐MOF as the carrier. This dual‐mode sensing platform shows great promise as a rapid, sensitive, and accurate tool for point‐of‐care testing and clinical diagnosis of nucleic acids of monkeypox virus and other infectious pathogens.

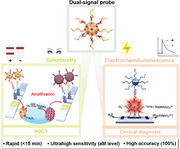

## 1 Introduction

Historically, human monkeypox (Mpox) was endemic to Africa (1970–2021). However, a surge in the number of Mpox cases outside Africa occurred in 2022.^[^ ^1^, ^2^ ^]^ To date, >90 000 positive cases have been reported in over 100 countries and regions worldwide (https://worldhealthorg.shinyapps.io/mpx_global). Despite the steady decline in the number of patients newly testing positive, real‐time surveillance remains critical in controlling the spread of Mpox, particularly in Africa and Asia, where infection rates are continuously increasing. Furthermore, early detection, isolation, and treatment are crucial in preventing its spread.^[^ ^3^ ^]^ Thus, biosensing platforms for rapid, ultrasensitive, and early diagnosis are urgently needed to prevent its spread and protect public health. Currently, quantitative polymerase chain reaction (qPCR) is commonly used to diagnose Mpox. However, qPCR is a multi‐temperature‐controlled process of nucleic acid amplification that is time‐consuming (1–2 h), requires expensive equipment (real‐time fluorescence PCR system, ≈$5000) and skilled technicians, are not available in some resource‐limited settings.^[^ ^4^ ^]^ To overcome the disadvantage of relying on expensive equipment, isothermal amplification assays, such as loop‐mediated isothermal amplification and recombinase polymerase amplification, have emerged as valuable tools for monkeypox virus (MPXV) detection.^[^ ^5^, ^6^, ^7^ ^]^ However, they are susceptible to amplification interference, complex primer design, time‐consuming protocols, elevated false positive rates, and stringent temperature control requirements, which hinder the broad application for Mpox diagnosis.^[^ ^8^ ^]^ Thus, the demand for a rapid, low‐cost, and convenient nucleic acid detection strategy toward Mpox has rapidly increased.

Colorimetric lateral flow assay (LFA) is one of the most widely used point‐of‐care testing (POCT) sensing platform for its advantages of rapidity, low cost, simplicity, and no need for professional skills, which enables on‐site qualitative visualization with the naked eye owing to its pronounced color change.^[^ ^9^, ^10^, ^11^, ^12^ ^]^ However, conventional gold nanoparticles based LFA (AuNPs‐LFA) is limited by its low sensitivity. What's more, because of its visual characteristics, LFA is only suitable for rapid on‐site screening and cannot be used for quantitative detection and accurate diagnosis, hindering its extensive applications.^[^ ^13^, ^14^ ^]^ In contrast, electrochemiluminescence (ECL) has received considerable research attention for its use in quantitative and accurate diagnosis owing to its high signal‐to‐noise ratio, and high sensitivity. For example, Wei et al. reported a self‐enhanced ECL chip for sensitive determination of SARS‐CoV‐2 with a portable assay setup.^[^ ^15^ ^]^ Nikolaou et al. presented a novel ECL molecular sensor with simple and portable instrumentation for ultrasensitive PCR‐free sensing of Hepatitis B Virus.^[^ ^16^ ^]^ Although their ECL signal detection equipment has been simplified, they still cannot be applied to on‐site screening and POCT. Therefore, designing ultrasensitive sensing platform integrating multiple scenarios of on‐site POCT screening and accurate quantification is immensely important for diagnosing Mpox.

The dual‐mode detection strategy is a more accurate sensor construction approach with a wider response range of application scenarios than single‐mode diagnostic techniques,^[^ ^17^, ^18^, ^19^ ^]^ and has two advantages: i) the advantages of each mode are preserved, resulting in a broader range and application scenarios,^[^ ^20^ ^]^ and ii) the accuracy and reliability are improved via the verification and comparison of the two signals.^[^ ^21^ ^]^ Several dual‐mode sensing platforms based on dual signals have been recently reported.^[^ ^22^, ^23^, ^24^, ^25^ ^]^ ECL methods combined with colorimetric assays are useful for establishing novel biosensing platforms as they not only enable simple semi‐quantitative visualization with the naked eye before accurate determination but also facilitate subsequent simple and rapid quantitative ECL analysis.^[^ ^26^ ^]^ Although various colorimetric and ECL dual‐mode biosensors have been investigated, the majority apply two completely independent signal probes (e.g., a nanozyme‐based catalyst and an individual ECL probe), to produce dual‐response signals, resulting in tedious sensing constructions.^[^ ^27^ ^]^ Thus, designing a reasonable single‐component dual‐functional signal probe, that can be simultaneously used as a colorimetric catalyst and ECL indicator for the dual‐mode sensing platforms is of great clinical significance.

**Figure 1:**
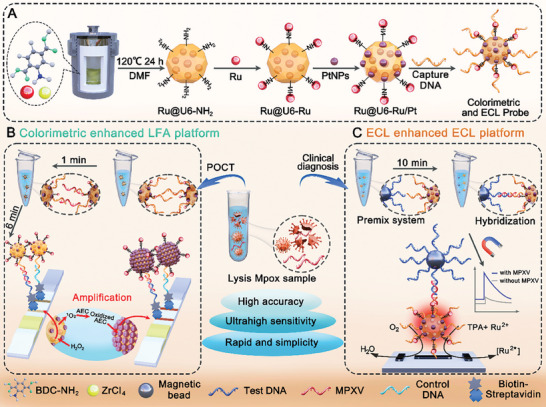
A) Schematic illustration of synthesis of Ru@U6‐Ru/Pt as the dual‐signal probe. B) Schematic illustration of colorimetric enhanced Ru@U6‐Ru/Pt NPs based LFA. C) Schematic illustration of Ru@U6‐Ru/Pt NPs‐based ECL sensing platform.

Considering the above‐mentioned factors, colorimetric and ECL enhancement dual‐mode biosensing platforms based on multifunctional nanoprobes Ru@U6‐Ru/Pt NPs were proposed for rapid, semi‐quantitative, and quantitative detection of monkeypox virus (MPXV) nucleic acid in different scenarios with two innovation points (**Figure** **1**). First, a dual‐signal probe Ru@U6‐Ru/Pt NPs was constructed by integrating double‐layered ECL luminophores and peroxidase‐like nanozyme PtNPs using Zr‐MOF (UiO‐66‐NH_2_) as the carrier (Figure 1A). To the best of our knowledge, this is the first to report of the superior performance of Ru@U6‐Ru/Pt NPs‐based dual‐signal tags into colorimetric and ECL dual‐mode sensing platforms. Second, we systematically analyzed the sensing range of the proposed dual‐mode sensing platforms and their sensitivity for MPXV detection. The colorimetric mode can be used as a rapid on‐site screening and POCT tool for Mpox without relying on specialized equipment. The ECL mode can support ultrasensitive and quantitative monitoring of Mpox in health‐care settings. The selectivity, accuracy, and practical detection capability of the proposed dual‐mode sensing platforms were also demonstrated in complex clinical samples. We aimed to provide a promising strategy for on‐site and accurate monitoring of Mpox, particularly in some resource‐limited settings.

## 2 Results

### 2.1 Principle of the Dual‐Mode Sensing Platforms

In this study, MPXV was used as a model target for colorimetric and ECL dual‐mode sensing platforms, which rely on a sandwich format without nucleic acid amplification (Figure 1). The clinical sample was initially heated at 95 °C for 5 min to release nucleic acid.

For the colorimetric Ru@U6‐Ru/Pt NPs‐based LFA platform, in the presence of the target, the target was first captured by Ru@U6‐Ru/Pt‐DNA nanoprobes to form the nanoprobes‐target complex via Watson‒Crick base pairing. The complex was then transferred onto the sample pad of the test strip for subsequent hybridization. Through capillary effect, the complex was further captured by the test DNA fixed on the T line to form a sandwich‐type complex owing to the second DNA hybridization reaction, resulting in a clear brown band from the accumulation of Ru@U6‐Ru/Pt NPs. Excess nanoprobes reacted with control DNA on the C line and a second brown band appeared. In the absence of the target, a sandwich‐type complex could not be generated on the T line; only Ru@U6‐Ru/Pt‐DNA nanoprobes directly reacted with the control DNA on the C line, resulting in only one brown band. Encouraged by the peroxidase (POD)‐like activity of the Ru@U6‐Ru/Pt NPs, the test strip was immersed into a mixture of 3‐amino‐9‐ethylcarbazole (AEC) and H_2_O_2_ for colorimetric signal amplification after immigration. The colorimetric signal intensity of the T line was substantially amplified by the catalytic deposition of insoluble brownish‐red products on Ru@U6‐Ru/Pt NPs, which effectively transduced and amplified the undetectable target‐binding molecular events, thus improving the detection sensitivity of LFA.

The Ru@U6‐Ru/Pt NPs‐based ECL platform, an amplification‐free, and rapid detection platform, included hybridization, magnetic beads enrichment, and ECL signal output. First, the lysed sample was added to the ECL sensing solution containing the capture probe (MB‐SA‐biotin‐DNA) and the signal probe (Ru@U6‐Ru/Pt‐DNA), mixed thoroughly, and reacted for 10 min. In the presence of a target, a sandwich‐type complex was obtained by trapping DNA with the capture probe and the signal probe via the base complementary base pairing principle. After magnetic separation, enrichment, and purification, the sandwich‐type complex was transferred to paper‐based bipolar electrodes and reacted with the co‐reagent tripropylamine (TPA) to obtain ECL signals. In contrast, sandwich‐type complexes could not form in the absorbance of the target, resulting in no ECL signals. Under the action of voltage, the formation mechanism of ECL signals was as follows:^[^ ^28^ ^]^

(1) $$Ru^{2+}\;-\;e^{-}\;\to Ru^{3+}$$

(2) $$\mathrm{TPA}\;-\;e^{-}\;\to \;\mathrm{TP}A^{\cdot +}$$

(3) $$\mathrm{TP}A^{\cdot +}\;-\;H^{+}\;\to \;{\mathrm{TPrA}}^{\cdot }$$

(4) $${\mathrm{TPA}}^{\cdot }\;+\;Ru^{3+\cdot }\;\to \;Ru^{2+*}\;+\;\mathrm{Products}$$

(5) $$Ru^{2+*}\;\to \;Ru^{2+}\;+\;hv$$

### 2.2 Characterization of Ru@U6‐Ru/Pt NPs

**Figure 2:**
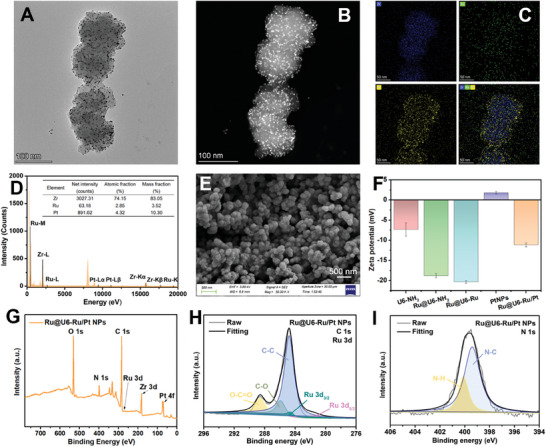
A) TEM image and B) HAADF‐STEM image of Ru@U6‐Ru/Pt NPs. C) EDX maps of Zr (blue), Ru (green), Pt (yellow), and their merged mapping. D) EDX spectrum of Ru@U6‐Ru/Pt NPs. E) SEM image of Ru@U6‐Ru/Pt NPs. F) The Zeta potential of U6‐NH_2_ NPs, Ru@U6‐NH_2_ NPs, Ru@U6‐Ru NPs, PtNPs, and Ru@U6‐Ru/Pt NPs. G) XPS survey scan of Ru@U6‐Ru/Pt NPs. High‐resolution of H) C1s and Ru 3d, and I) N 1s XPS spectra of Ru@U6‐Ru/Pt NPs.

As illustrated in Figure 1A, Ru@U6‐Ru/Pt NPs were synthesized in three steps. Briefly, Ru@UiO‐66‐NH_2_ (Ru@U6‐NH_2_) was prepared via a facile solvothermal method using 2‐aminoterephthalic acid (BDC–NH_2_) as the ligand, and ZrCl_4_ as the metal center, and the ECL luminophore Ru(bpy)_3_Cl_2_ was encapsulated. Using the abundant amino group of Ru@U6‐NH_2_, a considerable amount of the ECL luminophore Ru(dcbpy)_3_Cl_2_ was modified on Ru@U6‐NH_2_ through generation of amide bonds to obtain double‐layer Ru‐enriched Ru@U6‐Ru NPs. Finally, PtNPs were loaded on the Ru@U6‐Ru NPs as carriers using ultrasound to obtain Ru@U6‐Ru/Pt NPs. As shown in Figures S1A and S2A (Supporting Information), the U6‐NH_2_ NPs had a uniform regular octahedron structure with an average diameter of 91.9 ± 6.3 nm. After encapsulation of the first layer of Ru, the microstructure of the Ru@U6‐NH_2_ NPs changed into an icosahedron, and the average diameter changed to 93.7 ± 9.8 nm (Figures S1B and S2B, Supporting Information). However, the morphology and the average diameter of Ru@U6‐Ru NPs did not distinctly change after the second modification of Ru (Figures S1C and S2C, Supporting Information). Nonetheless, the solution color changed from light to dark orange (Figure S3A, Supporting Information). As displayed in Figure S2D (Supporting Information) and Figure **2**A, PtNPs were modified in Ru@U6‐Ru NPs, and Ru@U6‐Ru/Pt NPs could subsequently maintain the icosahedral morphology. The colors of the U6‐NH_2_, Ru@U6‐NH_2_, Ru@U6‐Ru, PtNPs, and Ru@U6‐Ru/Pt NPs were pink, light orange, dark orange, gray, and brown, respectively (Figure S3A, Supporting Information), indicating that these step‐wise synthesis products had been successfully prepared. High‐resolution transmission electron microscopy (HRTEM, Figure 2A) revealed that the obtained Ru@U6‐Ru/Pt NPs showed a clear icosahedral structure with an average diameter of 96.9 ± 5.9 nm. High‐angle annular dark field‐scanning electron microscope (HAADF‐STEM, Figure 2B) showed that high‐density PtNPs with an average size of 2.7 ± 0.4 nm (Figure S2D, Supporting Information) were embedded into Ru@U6‐Ru NPs to prepare Ru@U6‐Ru/Pt NPs owing to the loose porous structure of U6‐NH_2_ NPs, suggesting successful loading of PtNPs. The spatial‐arrangement of Zr, Ru, and Pt in the Ru@U6‐Ru/Pt NPs was examined using energy dispersive X‐ray (EDX) elemental mapping. The resulting elemental maps (Figure 2C) showed that Zr, Ru, and Pt were evenly distributed. EDX analysis (Figure 2D) further suggested the presence of Zr, Ru, and Pt in Ru@U6‐Ru/Pt NPs. Moreover, the calculated result of inductively coupled plasma‒mass spectrometer (ICP‐MS) results indicated that the Zr, Ru, and Pt concentrations were 411.4, 1.1, and 50.9 mg L^−1^, respectively, with an element content ratio of ≈374.0:1.0:46.3. The scanning electron microscopy image (SEM, Figure 2E) illustrated that Ru@U6‐Ru/Pt NPs showed a clear icosahedral structure with a rough surface. Zeta potential results are presented in Figure 2F. The Zeta potential of U6‐NH_2_ NPs was −7.3 mV and decreased to −18.8 mV after the first encapsulation of Ru. The Zeta potential of Ru@U6‐Ru NPs decreased to −20.3 mV after the second modification of Ru. After introduction of PtNPs, the Zeta potential of Ru@U6‐Ru/Pt NPs increased to −11.1 mV for the positive potential of PtNPs. These data provided conclusive evidence that Ru@U6‐Ru/Pt NPs were successfully prepared.

The compositions of the materials throughout the synthesis process were analyzed using X‐ray photoelectron spectroscopy (XPS). The full scan spectra of Ru@U6‐Ru/Pt NPs (Figure 2G) showed the presence of C, O, N, Zr, Ru, and Pt in Ru@U6‐Ru/Pt NPs. The Zr 3d spectra (Figure S4A, Supporting Information) of Ru@U6‐Ru/Pt NPs could be divided into two peaks of Zr 3d_3/2_ and Zr 3d_5/2_, featured at 184.6 and 182.2 eV,^[^ ^29^ ^]^ respectively and the binding energy of O 1s was 532 eV (Figure S4B, Supporting Information), suggesting that the Zr‐based metal‐organic framework (U6‐NH_2_) was successfully prepared. Regarding the C 1s high‐resolution XPS spectrum (Figure 2H), three peaks at 288.7, 285.9, and 284.8 eV were ascribed to O─C═O, C─O, and C─C,^[^ ^30^ ^]^ respectively. In addition, the emergence of two new peaks at 280.5 and 284.6 eV, with an energy gap of 4.1 eV, resulted from Ru 3d_5/2_ and Ru 3d_3/2_, respectively.^[^ ^30^ ^]^ The peaks appearing at 463.5 and 488.5 eV were attributed to Ru 3p_3/2_ and Ru 3p_1/2_, respectively (Figure S4C, Supporting Information).^[^ ^31^ ^]^ The XPS spectrum of N 1s displayed two peaks featured at 400.1 eV (N─H) and 399.4 eV (N─C), confirming the presence of a pyridine group derived from Ru,^[^ ^32^ ^]^ and indicating successful introduction of the luminescent Ru molecule into U6‐NH_2_ (Figure 2I). Regarding the Pt 4f high‐resolution XPS spectrum (Figure S4D, Supporting Information), the stronger pair at 70.9 (4f_7/2_) and 74.2 (4f_5/2_) eV verified the existence of metallic Pt^0^, and the weaker pair at 71.7 (4f_7/2_) and 75.6 (4f_5/2_) eV was attributed to Pt^2+^ species.^[^ ^33^ ^]^ The X‐ray diffraction (XRD) patterns exhibited three characteristic peaks at 7.3, 8.4, and 26, corresponding to the (111), (200), and (531) crystal planes,^[^ ^32^ ^]^ respectively (Figure S4E, Supporting Information). After modification of Ru and interposition of PtNPs, Ru@U6‐NH_2_, Ru@U6‐Ru, and Ru@U6‐Ru/Pt NPs exhibited highly similar crystal structures to those of U6‐NH_2_ NPs, implying that the skeleton of U6‐NH_2_ NPs was well preserved during the functionalized process. The Fourier Transform infrared spectroscopy (FTIR) spectra of U6‐NH_2_, Ru@U6‐NH_2_, Ru@U6‐Ru, and Ru@U6‐Ru/Pt NPs displayed similar peaks (Figure S4F, Supporting Information). The asymmetric vibration of the Zr─O bond produced two typical vibration bands at 670 and 740 cm^−1^,^[^ ^34^ ^]^ The peaks at 580 and 1420 cm^−1^ were derived from the stretching vibration of the carboxylic C═O bond and the symmetric vibration of the C─C bond in the benzene ring, respectively. This result suggested that the main functional groups in the BDC─NH_2_ were retained during the construction of Ru@U6‐NH_2_ NPs, Ru@U6‐Ru NPs, and Ru@U6‐Ru/Pt NPs.

### 2.3 Catalytic Property of Ru@U6‐Ru/Pt NPs

**Figure 3:**
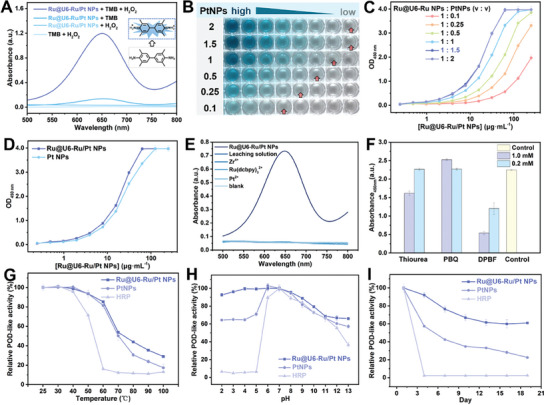
A) The visible absorption spectra of the mixture solution of Ru@U6‐Ru/Pt NPs+TMB+H_2_O_2_, Ru@U6‐Ru/Pt NPs+TMB, Ru@U6‐Ru/Pt NPs+H_2_O_2_, TMB+H_2_O_2_. B) Color development photos of Ru@U6‐Ru/Pt NPs with different PtNPs load ratios (0.1–2). C) The absorbance at 450 nm of Ru@U6‐Ru/Pt NPs with different PtNPs load ratios (0.1–2). D) Comparison of color development ability of Ru@U6‐Ru/Pt and PtNPs at the same concentration. The mechanism studies of POD‐like activity of Ru@U6‐Ru/Pt NPs: E) Zr^4+^, Ru(dcbpy)_3_^2+^ and Pt^2+^ ions leaching, F) the absorbance at 450 nm of the Ru@U6‐Ru/Pt NPs+TMB+H_2_O_2_ catalytic system with and without ROS scavengers. Notes: thiourea was used for scavenging •OH, 1,3‐diphenylisobenzofuran (DPBF) was chosen for scavenging ^1^O_2_, and p‐benzoquinone (PBQ) was utilized to capture O_2_^•−^. Comparison of different types of colorimetric stability of Ru@U6‐Ru/Pt NPs, free PtNPs, and HRP. G) Thermal stability. H) Chemical stability. I) Storage stability at 25 °C. The error bars represent the standard deviation (*n* = 3).

To investigate the colorimetric signal amplification ability, the catalytic property of Ru@U6‐Ru/Pt NPs was evaluated with the oxidation of TMB with H_2_O_2_. Ru@U6‐Ru/Pt NPs+H_2_O_2_ and TMB+H_2_O_2_ groups showed little absorption at 652 nm, whereas the Ru@U6‐Ru/Pt NPs+TMB group exhibited weak absorbance at 652 nm owing to oxidase‐like activity (**Figure** **3**A).^[^ ^33^ ^]^ The Ru@U6‐Ru/Pt NPs+TMB+H_2_O_2_ groups showed strong absorbance at 652 nm, originating from the peroxidase‐like (POD‐like) activity of Ru@U6‐Ru/Pt NPs, which was stronger than the absorbance generated from oxidase‐like activity. The POD‐like activity of Ru@U6‐Ru/Pt NPs was primarily derived from the PtNPs. Therefore, the amount of modified PtNPs determined the catalytic capacity of Ru@U6‐Ru/Pt NPs. As displayed in Figure 3B,C, after addition of H_2_O_2_ and TMB, the solution color and absorbance at 450 nm gradually darkened and increased with increasing amounts of PtNPs, reaching saturation at a load ratio of 1:1.5 (Ru@U6‐Ru NPs:PtNPs, v/v), indicating this value as the optimal load ratio of Ru@U6‐RuNPs to PtNPs. As displayed in Figure 3D, Ru@U6‐Ru/Pt NPs showed better catalytic activity than free PtNPs at the same amount of PtNPs, indicating that the aggregation of PtNPs with Ru@U6‐Ru NPs as carriers might have improved the catalytic property.^[^ ^35^, ^36^, ^37^ ^]^

An ions leaching experiment was performed to explore the POD‐like catalysis mechanism of Ru@U6‐Ru/Pt NPs.^[^ ^38^ ^]^ The concentrations of the analyzed metal ion solutions were the same as those of the leaching solution. Compared with that in the reaction solution of Ru@U6‐Ru/Pt NPs, the absorbance intensities of the metal ion solution and leaching solution were similar to those in the blank group (Figure 3E), indicating that the POD‐like activity of Ru@U6‐Ru/Pt NPs was attributable to itself rather than to ion leakage. Reactive oxygen species (ROS), such as •OH, ^1^O_2_, and O_2_^•−^, played a major role in the catalytic oxidation process of peroxidase mimics. Therefore, a series of ROS scavengers were used to scavenge the ROS. ^1^O_2_ was the main intermediate of the catalytic reaction (Figure 3F). A possible catalytic mechanism is the decomposition of H_2_O_2_ to generate ^1^O_2_ in the presence of Ru@U6‐Ru/Pt NPs, which then oxidizes TMB to generate blue oxTMB.^[^ ^39^ ^]^

To further study the POD‐like activity of Ru@U6‐Ru/Pt NPs, steady‐state kinetic assays were conducted by changing the TMB concentration to an invariable H_2_O_2_ concentration and vice versa (Figure S5, Supporting Information). A lower Michaelis constant (*Km*) value indicated a stronger affinity between the nanozyme and substrate. From the Michaelis‒Menten curves and Lineweaver‒Burk plots, the *Km* of Ru@U6‐Ru/Pt NPs for TMB and H_2_O_2_ was 0.08 and 6.87 mM, respectively (Table S2, Supporting Information). The *Km* of Ru@U6‐Ru/Pt NPs for TMB was lower than that for HRP (0.43 mM), indicating higher affinity for TMB than for HRP. However, the *Km* for H_2_O_2_ was higher than that for horseradish peroxidase (HRP, 3.70 mM), indicating that the higher the H_2_O_2_ concentration, the higher the Ru@U6‐Ru/Pt NPs activity. Moreover, the *Km* of Ru@U6‐Ru/Pt NPs was lower than that of most other PtNPs‐modified metal‐organic frameworks nanozymes (Table S2, Supporting Information), suggesting that Ru@U6‐Ru/Pt NPs exhibited superior POD‐like activity. In particular, the *Km* of Ru@U6‐Ru/Pt NPs was 2.5‐ and 1.7‐fold lower than that of free PtNPs for TMB and H_2_O_2_, respectively, indicating that the aggregation of PtNPs with Ru@U6‐Ru NPs as carriers to form Ru@U6‐Ru/Pt NPs might have enhanced the POD‐like activity.^[^ ^35^, ^36^, ^37^ ^]^

Considering that the stability of the nanoprobe was critical for the repeatability and accuracy of the test strip detection results, the thermal‐, pH‐, and long‐term storage stabilities of the Ru@U6‐Ru/Pt NPs were explored by performing catalytic oxidation of the TMB substrate with H_2_O_2_. Free HRP and PtNPs were treated similarly for comparison. Figure 3G demonstrated that the catalytic activity of the Ru@U6‐Ru/Pt NPs decreased from 100% to 85.3% when the temperature increased from 25 to 60 °C, and that of free HRP decreased from 100% to 16.1%, suggesting that the catalytic activity of Ru@U6‐Ru/Pt NPs was significantly higher than that of free HRP at different temperatures. The catalytic activity of Ru@U6‐Ru/Pt NPs showed negligible changes at pH 2–7 and then decreased to 66.0% as the pH increased from 7 to 13 (Figure 3H). However, the catalytic activity of free HRP decreased to 4.8% at pH 2−7, demonstrating that the Ru@U6‐Ru/Pt NPs were more stable than free HRP at pH 2−7. The catalytic activity of Ru@U6‐Ru/Pt NPs decreased (to 98%)less than that of free HRP (to 2.3%) after being stored at 25 °C for 4 days, and the catalytic activity of Ru@U6‐Ru/Pt NPs remained at 61.0% after being stored at 25 °C for 20 days, indicating that Ru@U6‐Ru/Pt NPs had improved storage stability (Figure 3I). Moreover, Ru@U6‐Ru/Pt NPs showed improved thermal, pH, and long‐term storage stabilities compared to those of free PtNPs, suggesting that the aggregation of PtNPs with Ru@U6‐Ru NPs as carriers to form Ru@U6‐Ru/Pt NPs enhanced the stability of POD‐like activity. Overall, the excellent thermal, pH, and long‐term storage stabilities made Ru@U6‐Ru/Pt NPs suitable as multifunctional nanoprobe for enhancing the LFA detection performance.

### 2.4 Enhanced ECL Activity of Ru@U6‐Ru/Pt NPs

**Figure 4:**
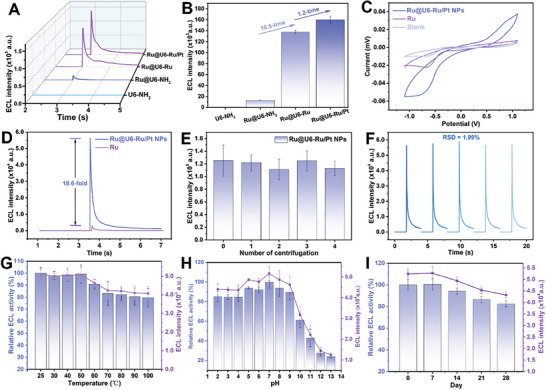
A) The ECL spectra and B) the comparison of ECL intensity of U6‐NH_2_ NPs, Ru@U6‐NH_2_ NPs, Ru@U6‐Ru NPs, PtNPs, and Ru@U6‐Ru/Pt NPs. C) Cyclic voltammetry profiles and D) the ECL intensity of Ru(dcbpy)_3_Cl_2_ and Ru@U6‐Ru/Pt NPs with the same concentration of Ru(dcbpy)_3_Cl_2_. E) The ECL intensity of Ru@U6‐Ru/Pt NPs before and after the centrifugation. F) The stability of ECL intensity of Ru@U6‐Ru/Pt NPs. Different types of ECL stability of Ru@U6‐Ru/Pt NPs. G) Thermal stability. H) Chemical stability. I) Storage stability at 25 °C. Error bars denote the standard deviation (*n* = 3).

High‐efficiency enrichment of ECL luminophores is one of the strategies to improve the sensitivity of ECL sensors. In this study, a Zr‐based metal‐organic framework (U6‐NH_2_ NPs) with a large specific surface area and rich amino groups was selected as a carrier to enrich ECL luminophores for signal amplification. The ECL intensity of Ru@U6‐NH_2_ NPs encapsulated with the first layer of Ru was ≈1.3×10^4^ a.u., whereas that of the carrier U6‐NH_2_ NPs was ≈700 a.u., indicating that Ru was successfully encapsulated in the U6‐NH_2_ NPs and that the first signal enrichment was achieved (**Figure** **4**A,B). The ECL intensity of Ru@U6‐Ru NPs modified with double layers of Ru was ≈1.37 × 10^5^ a.u., which was 10.5 times higher than that of Ru@U6‐NH_2_ NPs encapsulated with a single layer of Ru, demonstrating that the second signal amplification was also successful. After the introduction of PtNPs, which can promote electron transfer efficiency, the ECL intensity of Ru@U6‐Ru/Pt NPs increased to 1.6 × 10^5^ a.u., achieving the third signal amplification. Electrochemical and ECL measurements were performed to further investigate the signal amplification of Ru@U6‐Ru/Pt NPs. Both Ru and Ru@U6‐Ru/Pt NPs exhibited oxidation peaks at 1.1 V, and the current of Ru@U6‐Ru/Pt NPs was significantly higher than that of Ru at the same Ru concentration (Figure 4C). This enhancement was further demonstrated by ECL measurements. The ECL intensity of the Ru@U6‐Ru/Pt NPs was 18.6‐fold higher than that of equal concentrations of free Ru (Figure 4D), indicating that the aggregation of Ru with U6‐NH_2_ NPs as the carrier and introduction of PtNPs might have improved the ECL intensity for efficient signal amplification.

Ru@U6‐Ru/Pt NPs were centrifuged four times at high speed, the precipitate was dispersed in the original volume of ultrapure water to obtain a suspension, and the ECL intensity was tested. The ECL intensity of the centrifuged Ru@U6‐Ru/Pt NPs suspension was similar to that of the untreated Ru@U6‐Ru/Pt NPs, indicating that there was no leakage of luminescent molecules during the high‐speed centrifugation process (Figure 4E). The ECL signal stability of Ru@U6‐Ru/Pt NPs was evaluated by consecutive measurements (Figure 4F). The ECL signal remained stable with relative standard deviations (RSDs) of 1.99%, indicating the high signal stability of Ru@U6‐Ru/Pt NPs, which has great potential for application in the construction of Ru@U6‐Ru/Pt NPs‐based ECL biosensors. The ECL activity stability is critical for the accuracy and repeatability of the platform. Therefore, the thermal, pH, and long‐term storage stabilities of the Ru@U6‐Ru/Pt NPs were evaluated. The ECL activity of the Ru@U6‐Ru/Pt NPs decreased from 100% to 79.7% when the temperature increased from 25 to 100 °C (Figure 4G). The ECL activity of Ru@U6‐Ru/Pt NPs was maintained at 84.5–100% at pH 2−9 and substantially decreased at pH 10−13 (Figure 4H), which may be due to the breaking of the amide bond at strong alkalinization, resulting in the separation of the second layer of Ru connected by the amide bond from the carrier Zr‐MOF. The ECL activity of the Ru@U6‐Ru/Pt NPs was maintained at 82.6% after storage at 25 °C for 28 days (Figure 4I), indicating that the excellent long‐term storage stabilities made Ru@U6‐Ru/Pt NPs suitable as the nanoprobe for construction of the Ru@U6‐Ru/Pt NPs based ECL platform.

### 2.5 Characterization of Capture and Signal Probes

The capture probe was the conjugation of streptavidin magnetic beads and biotin‐modified DNA (test DNA) via a reaction between streptavidin and biotin, and the signal probe was the conjugation of Ru@U6‐Ru/Pt NPs and sulfhydrated DNA (capture DNA) through the formation of Pt‐S bonds. Fluorescein FAM‐modified test DNA and capture DNA were utilized to characterize the capture and signal probes, respectively. An absorption peak at 519 nm was seen before the conjugation, whereas there was no absorption peak at 519 nm in the supernatant after conjugation, indicating that the test DNA and capture DNA were modified on the streptavidin magnetic beads and Ru@U6‐Ru/Pt NPs, respectively (Figure S6A,B, Supporting Information). Zeta potentials of test DNA and streptavidin magnetic beads were −2.4 and −7.5 mV, respectively. After the conjugation, the Zeta potentials of the capture probe turned into −18.5 mV (Figure S6C, Supporting Information), further indicating that the capture probe was successfully prepared.^[^ ^40^, ^41^ ^]^ Similarly, the potential changed substantially after the signal probe was formed (Figure S6D, Supporting Information), further indicating successful preparation of the signal probe.

### 2.6 Detection Performance of the Ru@U6‐Ru/Pt NPs‐Based LFA

To obtain appropriate performance of the general Ru@U6‐Ru/Pt NPs based LFA, several experimental factors were thoroughly investigated, including the volume of capture DNA for the conjugation process, the volume of Ru@U6‐Ru/Pt‐DNA probe, the migration time, the volume of target, and the volume of NaCl solution. Two indicators were selected to confirm the optimal parameters: relative signal intensity (expressed using the S/N value) and signal uniformity (expressed using the T_+_/C_+_ value). The S and N values represented the optical signal intensity on the T line for MPXV ssDNA (1 µM) and ultrapure water, respectively. Similarly, T_+_ and C_+_ denoted as the optical signal intensities on the T and C line for MPXV ssDNA (1 µM), respectively. The T_+_/C_+_ value ratio was ≈1.0, indicating that the optical signals of the T and C lines were uniform. Therefore, the optimal volume of capture DNA for the conjugation process, volume of Ru@U6‐Ru/Pt‐DNA probe, migration time, volume of target, and volume of NaCl solution were 20 µL, 8 µL, 6 min, 2 µL, 10 µL, and 1 min, respectively (Figures S7 and S8, Supporting Information). For optimal performance of the colorimetric enhanced Ru@U6‐Ru/Pt NPs‐based LFA, analytical parameters including loading modes and the concentration of AEC and H_2_O_2_ solution were optimized. As seen in Figure S9 (Supporting Information), the loading mode of total immersion and AEC and H_2_O_2_ solution concentration of 12.5 mM exhibited strong signal intensity (high S/N value) and enhancement (high ΔT intensity). ΔT intensity was defined as the difference in the optical signal intensity on the T line after subtracting the background optical signal intensity before and after color enhancement with AEC and H_2_O_2_ solution.

**Figure 5:**
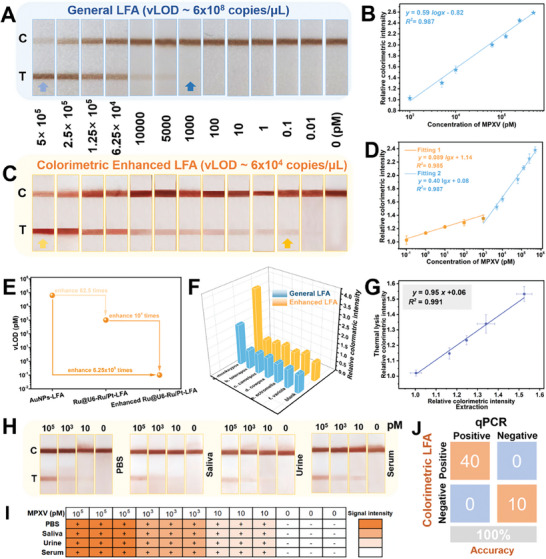
Analytical performance of the prepared Ru@U6‐Ru/Pt NPs based LFA test strips for the detection of MPXV. A,C) Photographs of the test strips in response to different concentrations of MPXV A) before and C) after AEC catalytic amplification. B,D) Calibration curves of the T line intensity before and after AEC catalytic amplification, respectively. E) Comparison of *v*LOD and the sensitivity among AuNPs‐LFA, general Ru@U6‐Ru/Pt NPs based LFA, and colorimetric enhanced Ru@U6‐Ru/Pt NPs based LFA. F) Selectivity of the Ru@U6‐Ru/Pt NPs based LFA test strips. (G) Correlation between thermal lysis samples and extracted samples detected by Ru@U6‐Ru/Pt NPs based LFA test strips. H) Photographs and I) qualitative results of the test strips for the recovery of MPXV from spiked samples by Ru@U6‐Ru/Pt NPs based LFA test strips. All experiments were performed in triplicate. J) Evaluation of the sensitivity and specificity of Ru@U6‐Ru/Pt NPs based LFA compared with standard qPCR using a confusion matrix.

A rapid, instrument‐independent, and on‐site Ru@U6‐Ru/Pt NPs‐based LFA was constructed for POCT in private residences. Using MPXV as the target, the detection performance of general and colorimetric enhanced LFA was investigated by testing various concentrations of the synthetic standard MPXV ssDNA (0–5 × 10^5^ pm). With increasing MPXV concentration, the color of the T line gradually deepened, indicating that the nanozyme‐catalyzed deposition amplification did not alter the concentration‐dependent quantitative relationship (**Figure** **5**A,C). The visual sensing range for MPXV analysis before and after catalysis ranged from 10^3^ to 5 × 10^5^ pM and 0.1 to 5 × 10^5^ pM, respectively. The visual limit of detection (*v*LOD) was defined as the minimum MPXV ssDNA concentration, at which the color of the T line was significantly higher than that of the negative strip.^[^ ^41^ ^]^ The *v*LODs of the proposed LFA before and after catalysis were 1000 (6 × 10^8^ copies·µL^−1^) and 0.1 pm (6 ×10^4^ copies·µL^−1^), respectively, being 62.5‐ and 6.25 × 10^5^‐fold lower than those of conventional AuNPs‐based LFA (Figures S10A, Supporting information; Figure 5E). For semi‐quantitative analysis, Figure 5B,D showed the calibration curves obtained by plotting the relative colorimetric intensity value between the experimental and blank groups of the T line against the MPXV ssDNA concentration before and after signal amplification. The semi‐LOD values (relative colorimetric intensity of the blank samples plus three times of the standard deviation) before and after signal amplification were 950 and 0.06 pM, respectively. The semi‐quantitative detection range for MPXV analysis before and after catalysis ranged from 10^3^ to 5 × 10^5^ pm (*R^2^* = 0.987) and 0.1 to 5 × 10^5^ pm (*R^2^* = 0.993 and 0.984), respectively. The colorimetric enhanced Ru@U6‐Ru/Pt NPs‐based LFA developed in this study was superior to the previously reported colorimetric LFA for detecting MPXV or other nucleic acid targets at the non‐amplification level with a lower LOD and shorter time. Although the amplification method achieved higher sensitivity, it required a longer time, which was not conducive for POCT, implying that the sensing nanoprobe Ru@U6‐Ru/Pt NPs with the amplification‐free strategy may offer a promising alternative to traditional AuNPs‐based LFA for improving detection sensitivity (Table S3, Supporting Information).

The specificity of the developed Ru@U6‐Ru/Pt NPs‐based LFA was investigated by analyzing several synthetic standard ssDNA of other orthopoxviruses, including taterapox, camelpox, cowpox, ectromelia, and variola, at a concentration of 10^5^ pm, while the concentration of MPXV was 10^4^ pm. As displayed in Figures 5F and Figure S10 (Supporting Information), both general and colorimetric enhanced Ru@U6‐Ru/Pt NPs‐based LFAs showed strong signal responses toward MPXV compared to those toward the blank control and other orthopoxviruses, implying high specificity of the proposed Ru@U6‐Ru/Pt NPs based LFA for specifically distinguishing MPXV from other orthopoxviruses. The accuracy and matrix effect of the Ru@U6‐Ru/Pt NPs‐based LFA was evaluated by determining the recovery of MPXV‐spiked samples in three types of artificial matrices including saliva, urine, and serum, at concentrations of 10, 10^3^, and 10^5^ pm, respectively. The brownish‐red color of the T line changed from shallow to dark with an increase of the MPXV ssDNA concentration and showed a signal intensity similar to that of the PBS group, indicating that the Ru@U6‐Ru/Pt NPs‐based LFA displayed a sensitive response to MPXV and had high tolerance to saliva, urine, and serum matrices (Figure 5H,I).

### 2.7 Analytical Evaluation of the Ru@U6‐Ru/Pt NPs Based ECL Platform

**Figure 6:**
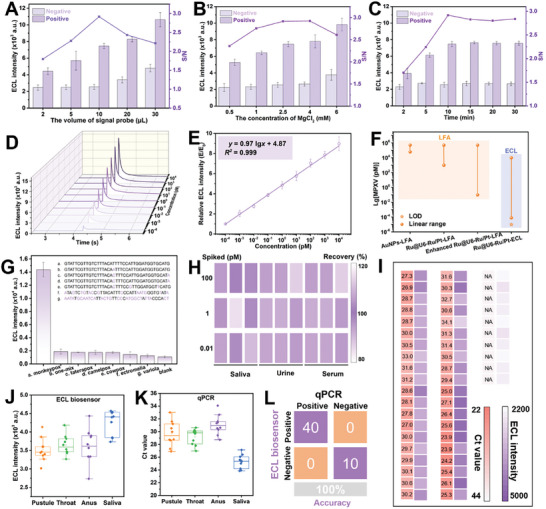
Optimization results of A) the volume of the signal probe, B) the concentration of MgCl_2_, and C) the incubation time of the Ru@U6‐Ru/Pt NPs based ECL biosensor. D) ECL responses of the Ru@U6‐Ru/Pt NPs based ECL biosensor toward different concentrations of MPXV from 10^−4^ to 10^4^ pm under the ECL mode. E) Calibration curve of the ECL responses with respect to MPXV concentration. F) Comparison of LOD and the linear range among colorimetric LFA and ECL developed in this work. G) Selectivity of the Ru@U6‐Ru/Pt NPs based ECL biosensor. H) Recovery results of spiked samples by Ru@U6‐Ru/Pt NPs based ECL biosensor. I) ECL responses of healthy clinical samples and MPXV‐positive samples collected from pustule, throat, anus, and saliva and detected by the Ru@U6‐Ru/Pt NPs‐based ECL biosensor. J) Ct value of MPXV‐positive samples collected from pustule, throat, anus, and saliva and detected by qPCR. K) Comparison of Ru@U6‐Ru/Pt NPs based ECL biosensor and commercial qPCR detected values for MPXV in clinical samples. L) Evaluation of the sensitivity and specificity of Ru@U6‐Ru/Pt NPs based ECL biosensor compared with standard qPCR using a confusion matrix.

A rapid, highly sensitive, and accurate quantitative Ru@U6‐Ru/Pt NPs‐based ECL platform was constructed, which is suitable for medical institutions such as community hospitals and medical centers. The detection performance of the Ru@U6‐Ru/Pt NPs based ECL platform was related to several factors, including the volume of the signal probe (Ru@U6‐Ru/Pt‐DNA), the concentration of MgCl_2_, and incubation time. Two indicators with signal intensity and an S/N value were selected to confirm the optimal parameter. The larger the S/N value and the greater the signal intensity, the better the performance of the Ru@U6‐Ru/Pt NPs based ECL platform. The optimal volume of the signal probe, concentration of MgCl_2_, and incubation time were 10 µL, 2.5 mm, and 10 min, respectively (**Figure** **6**A‒C). Under optimal conditions, MPXV standards at various concentrations (0‒10^4^ pm) were monitored using the Ru@U6‐Ru/Pt NPs‐based ECL platform. The ECL intensity gradually increased as the MPXV ssDNA concentration increased from 10^−4^ to 10^4^ pm (Figure 6D). Figure 6E showed a strong linear relationship between the relative ECL intensity (E/E_0_) and logarithm of MPXV ssDNA concentration in the range of 10^−4^ to 10^4^ pm with a linear regression equation of *y* = 0.97 *lgx* + 4.87, LOD of 10 aM (6 copies·µL^−1^) and correlation coefficient (*R^2^*) of 0.999. Additionally, the above results were compared with those of the aforementioned Ru@U6‐Ru NPs based LFA and recently published MPXV detection strategies (Figure 6F). Compared with existing MPXV detection assays, the Ru@U6‐Ru/Pt NPs based ECL platform showed a lower LOD and wider linear range with the shortest sensing time (Table S4, Supporting Information). In addition, the utilization of thermal lysis without extraction for the sample treatment made it easy‐to‐use, and time‐saving. Thus, it can be concluded that this Ru@U6‐Ru/Pt NPs based ECL platform presented superior performance for sensing MPXV. However, it has minimal potential for POCT detection because it relies on ECL analytical instruments. Conversely, the colorimetric enhanced Ru@U6‐Ru/Pt NPs based LFA developed in this study provides relatively weak sensitivity, however it could be used for POCT, which could offset the disadvantages of the Ru@U6‐Ru/Pt NPs based ECL platform. These two modes complemented each other to achieve accurate and rapid MPXV detection.

The specificity of this Ru@U6‐Ru/Pt NPs‐based ECL platform was investigated using five synthetic standard ssDNAs from other orthopoxviruses (i.e., taterapox, camelpox, cowpox, ectromelia, and variola). Only the assay for MPXV at 10^3^ pm generated a distinct ECL intensity, whereas no evident ECL intensity was observed for the other orthopoxviruses at 10^4^ pm, demonstrating the favorable selectivity of the ECL‐sensing mode for MPXV (Figure 6G). Moreover, the Ru@U6‐Ru/Pt NPs based ECL platform was used to monitor MPXV in spiked saliva, urine, and serum samples. The average recovery values (*n* = 3) were 87.1‒110.1%, within the allowable ranges, indicating that the ECL‐sensing mode showed favorable accuracy for MPXV detection (Figure 6H). These results confirmed that the Ru@U6‐Ru/Pt NPs‐based ECL platform could detect MPXV with high sensitivity (6 copies·µL^−1^), high efficiency (10 min), a wide sensing range (10^−4^ to 10^4^ pm), and excellent accuracy (87.1‒110.1%).

### 2.8 Clinical Application of Dual‐Mode Sensing Platforms

To simplify the workflow of the sample pretreatment, thermal lysis was utilized instead of the traditional nucleic acid extraction method. As shown in Figure S11A,B (Supporting Information) and Figure 5G, for general and colorimetric enhanced Ru@U6‐Ru/Pt NPs based LFAs, the signal intensity of the T line generated from thermal lysis samples was consistent with that of nucleic acid extraction samples, indicating that the simple and rapid thermal lysis method was suitable for Ru@U6‐Ru/Pt NPs based LFA. Similar results were obtained for the Ru@U6‐Ru/Pt NPs‐based ECL platform with a correlation coefficient of 0.998 between these two sample pretreatment methods (Figure S11C,D, Supporting Information), further demonstrating that the simpler and more rapid thermal lysis treatment method was also applicable to Ru@U6‐Ru/Pt NPs based ECL. In conclusion, compared with the multi‐step, time‐consuming (>20 min) operation of nucleic acid extraction, the thermal lysis treatment method not only had a short incubation time (5 min) but was also suitable for the dual‐mode sensing platforms developed in this study, indicating its superior potential for rapid MPXV detection.

To validate the reliability and practicability of the developed platform, 50 clinical samples—40 Mpox‐positive samples (10 pustule, 10 throat, 10 anus, and 10 saliva samples) and 10 Mpox‐negative samples from healthy volunteers were collected and monitored using the developed Ru@U6‐Ru/Pt NPs based dual‐mode sensing platforms alongside with the qPCR assays. qPCR with viral extraction required ≈2 h; conversely, dual‐mode sensing platforms with viral lysis required <15 min. No distinct band was seen on the T line of the above‐mentioned 40 Mpox‐positive samples detected using the general Ru@U6‐Ru/Pt NPs based LFA (Figure S12A, Supporting Information). However, weak bands appeared on the T line detected by the colorimetric enhanced Ru@U6‐Ru/Pt NPs based LFA, and the bands of the saliva samples were stronger than those collected from other parts of the body (Figure S12B, Supporting Information), indicating that signal amplification is necessary for clinical use. Samples were then analyzed using the Ru@U6‐Ru/Pt NPs based ECL platform (Figure S13A‒D, Supporting Information). Clear ECL signals in all Mpox‐positive samples and weak ECL signals in Mpox‐negative samples were observed (Figure 6J), revealing that the Ru@U6‐Ru/Pt NPs‐based ECL platform correctly identified all Mpox‐positive and Mpox‐negative samples with 100% accuracy (Figure 6L). Notably, saliva samples produced a stronger ECL signal intensity than those collected from other parts of the body, indicating that the virus was present in saliva at a higher level and inferring that using saliva samples could improve the accuracy rate of positive tests. Detection results were compared with those obtained using commercial qPCR analysis (Figure S13E−H, Supporting Information). The Ct values of the Mpox‐positive samples determined by qPCR assay ranged from 23.9 to 34.1 (Figure 6I). The Ct values of the saliva samples were lower than those of the pustule, throat, and anus samples (Figure 6K), indicating a higher MPXV concentration in saliva samples, which was similar to the results obtained using the Ru@U6‐Ru/Pt NPs based ECL platform. The results of the proposed Ru@U6‐Ru/Pt NPs based colorimetric enhanced LFA and ECL platform were 100% concordant with those of qPCR for all 50 clinical samples, indicating that the developed dual‐mode sensing platforms could serve as an alternative to traditional qPCR for rapid and accurate MPXV detection (Figures 5J and 6L).

## 3 Conclusion

In summary, flexible Ru@U6‐Ru/Pt NPs based colorimetric‐ECL dual‐mode platforms were proposed for rapid and ultrasensitive MPXV detection for POCT in private residences and quantitative analysis in medical institutions. The newly invented Ru@U6‐Ru/Pt NPs nanotags were constructed by integrating the double‐layered ECL luminophores and the POD‐like nanozyme PtNPs using Zr‐MOF (UiO‐66‐NH_2_) as carriers, generating strong ECL and colorimetric signals with excellent stability. With this nanotag, the dual‐mode platforms achieved rapid screening (colorimetric enhanced LFA) and quantitative analysis (ECL platform) of the MPXV nucleic acids within 15 min, with *v*LOD (by the naked eye) and LOD (by the small ECL device) as low as 0.1 pm and 10 am, respectively, which was superior to previously reported sensing platforms for MPXV or other nucleic acid targets. Moreover, the sensing performance of these dual‐mode platforms was validated using 50 clinical samples (40 Mpox‐positive samples from pustules, throat, saliva, and anus, and 10 Mpox‐negative samples), resulting in 100% concordance with the qPCR results. The developed Ru@U6‐Ru/Pt NPs‐based dual‐mode platforms are recommended for their ease of use, time‐saving capacity, and suitability for on‐site use, and could be served as an affordable, user‐friendly tool for the rapid monitoring of MPXV or other highly pathogenic viruses. The dual‐mode sensing platforms not only have complementary advantages but also further improve detection accuracy and sensing range. The novel integrated functional nanomaterials have the combined advantages of dual‐mode readout, high sensitivity, and high accuracy, suggesting their broad application prospects in the development of sensing probes. How to fully utilize the characteristics of nanomaterials and efficiently construct multifunctional nanomaterials to meet more needs is undoubtedly the hot spot and trend of current research and also the direction of future exploration.

## 4 Experimental Section

### Synthesis of Ru@U6‐Ru/Pt NPs

The nanotag of Ru@U6‐Ru/Pt NPs was synthesized by a three‐step process as previously reported with little modifications.^[^ ^42^ ^]^

### Synthesis of Ru@U6‐NH_2_ NPs

Briefly, a homogeneous solution containing ZrCl_4_ (48.2 mg), 2‐aminoterephthalic acid (BDC‐NH_2_, 39.2 mg), Ru(bpy)_3_Cl_2_ (6.5 mg), and N,N‐dimethylformamide (DMF, 18.0 mL) was obtained through ultrasonication. Then, DMF (12 mL) and acetic acid (300 µL) were added and stirred for 10 min. Finally, the above mixture was transferred to a Teflon‐lined autoclave and maintained at 120 °C for 24 h. After cooling to room temperature, the pink precipitate was suspended in ultrapure water (30 mL) after centrifugation at 7140 g for 10 min and washing with ethanol and ultrapure water.

### Synthesis of Ru@U6‐Ru NPs

First, the coupling reagents of N‐hydroxysuccinimide (10 mg mL^−1^, 38 µL) and 1‐ethyl‐3‐(3dimethylaminopropyl) carbodiimide hydrochloride (10 mg mL^−1^, 65 µL) were added into Ru(dcbpy)_3_Cl_2_ solution (1 mg mL^−1^, 2 mL) to activate the carboxyl groups of Ru(dcbpy)_3_Cl_2_, and then the mixture was stirred for 1 h at room temperature. Then, Ru@U6‐NH_2_ NPs (10 mL) were added and stirred for 8 h to combine the carboxyl groups of Ru(dcbpy)_3_Cl_2_ to form Ru@U6‐Ru NPs. Finally, Ru@U6‐Ru NPs were collected by centrifugation at 7140 g for 10 min and then suspended in ultrapure water (10 mL).

### Synthesis of Ru@U6‐Ru/Pt NPs

Synthesis of Ru@U6‐Ru NPs (10 mL) was mixed with PtNPs solution (15 mL) and then sonicated for 30 min. The brown precipitates were collected by centrifugation (7140 g, 10 min) after being washed with ultrapure water, and then suspended in 10 mL of ultrapure water. The final product was stored at 4 °C for further use.

### Preparation of the Ru@U6‐Ru/Pt‐DNA Nanoprobes

The sensing nanoprobes were prepared as previously reported with minor modifications.^[^ ^43^ ^]^ Briefly, the thiolated capture DNA (20 µm, 25 µL), which were pretreated with tris(2‐carboxyethyl)phosphine hydrochloride (10 mm) to reduce disulfide bonds, were introduced into Ru@U6‐Ru/Pt NPs (1 mL) solution and were shaken overnight for the conjugation to form the Ru@U6‐Ru/Pt‐DNA nanoprobes. After centrifugation (7140 g, 10 min) and washing with ultrapure water, the nanoprobes were suspended in 0.5 mL of ultrapure water and stored at 4 °C for further experiment.

### Fabrication of Ru@U6‐Ru/Pt NPs Based LFA

Ru@U6‐Ru/Pt NPs based LFA test strip was assembled according to the previous study.^[^ ^44^ ^]^ The test strip comprised five parts, including a sample pad, conjugate pad, NC membrane, absorbent pad, and PVC backing plate. The test line (T line) and control line (C line) on the NC membrane were acquired by separately spraying the streptavidin‐biotinylated test DNA conjugates and the streptavidin‐biotinylated control DNA conjugates, which were obtained by mixing an equal volume of the streptavidin solution (1 mg mL^−1^) and biotinylated DNA (100 µm) for 2 h at 25 °C. Then the above were assembled on the PVC backing plate as shown in Figure 1B. Finally, the prepared test strips were cut into pieces with a width of 4.0 mm.

To validate the sensitivity of this Ru@U6‐Ru/Pt NPs‐based LFA, the synthetic standard Mpox ssDNA (2 µL) with varying concentrations were added into the sensing solution containing Ru@U6‐Ru/Pt‐DNA nanoprobes (5 µL), running buffer (60 µL, 1.5% PEG 20 000, 1% fructose,1% BSA, and 1% Tween‐20), and NaCl solution (10 µL, 1 m). After incubation at room temperature for 1 min, the above mixture (60 µL) was loaded into the sample pad of the test strip. After a running time of 6 min, the reacted test strip was immersed in a commercial substrate solution containing the AEC substrate and H_2_O_2_ (12.5 mm) to conduct signal amplification by enzyme‐catalyzed deposition. After immersion for 1 min, the reacted test strips were directly subjected to qualitative and semi‐quantitative measurements without washing and drying. The photograph of test strips and the corresponding optical densities at T and C lines were recorded using an image analyzer (Amersham ImageQuant 800, Cytia, Sweden) and then analyzed with a software Image J, respectively.

### Construction of Ru@U6‐Ru/Pt NPs Based ECL Biosensor

Ru@U6‐Ru/Pt NPs based ECL biosensor was constructed based on the principle of complementary base pairing with the amplification‐free process. Briefly, the synthetic standard Mpox ssDNA (2 µL) with varying concentrations was added into the ECL sensing solution containing of signal probe (Ru@U6‐Ru/Pt‐DNA, 10 µL), capture probe (MB‐SA‐biotin‐DNA, 10 µL), MgCl_2_ solution (2.5 mm) and ultrapure water (200 µL). After shaking at room temperature for 10 min, the hybrid sandwich product was separated by magnet and resuspended in ultrapure water (30 µL) after being washed with ultrapure water. Afterward, tripropylamine (TPA, 150 µL) was added into the above resuspension. Finally, the mixture (15 µL) was transferred to the disposable paper‐based bipolar electrode, which was inserted in the portable ECL signal detection device with the working voltage of 20 V and an integral time of 10 s.

### Sample Analysis via Dual‐Mode Sensing Platforms

To assess the feasibility of dual‐mode sensing platforms for Mpox detection, artificial saliva, urine, and serum were used as matrices for spiking and recovery analysis. First, synthetic standard MPXV ssDNA was added to the above‐mentioned matrices to generate spiked samples at different concentrations. Then, the spiked solution was analyzed using dual‐mode sensing platforms. A total of 50 clinical samples (40 and 10 Mpox‐positive and ‐negative, respectively) were obtained from the Shenzhen Third People's Hospital, China. For analysis of clinical samples, nucleic acids were either extracted using the MagaBio plus virus DNA/RNA purification kit or released using heat treatment at 95 °C for 5 min.

### Statistical Analysis

The data calculated in the experiment were all presented as mean ± SD, and each data quantitative evaluation was repeated at least three times. All data were analyzed by Origin 2022.

## Conflict of Interest

The authors declare no conflict of interest.

## Supporting information

Supporting Information

## Data Availability

The data that support the findings of this study are available from the corresponding author upon reasonable request.
