# Supplementary material for: Zr‐MOF Carrier‐Enhanced Dual‐Mode Biosensing Platforms for Rapid and Sensitive Diagnosis of Mpox
Source: Adv Sci (Weinh). 2024 Aug 9;11(38):2405848. doi: 10.1002/advs.202405848 (PMC11481339; doi:10.1002/advs.202405848)
Supplement: Supplementary file 1 — Supporting Information [file ADVS-11-2405848-s001.docx]

Supporting Information

Zr-MOF Carrier-Enhanced Dual-Mode Biosensing Platforms for Rapid and Sensitive Diagnosis of Mpox

Huiyi Yang^†^, Judun Zheng^†^, Wei Wang^†^, Jingyan Lin^†^, Jingru Wang, Lunjing Liu, Wenjie Wu, Chengli Zhang, Mingxia Zhang*, Yu Fu*, Bin Yang*, Yuhui Liao*

**Content**

[Experimental Section 3](#_Toc171778191)

[Tables 6](#_Toc171778198)

[Figures 8](#_Toc171778199)

[References 18](#_Toc171778200)

# Experimental Section

## Reagents

All oligonucleotide sequences (listed in **Table S1**) used in this work were synthesized by Sangon Biotech Co., ltd (Shanghai, China). Zirconium chloride (ZrCl_4_), chloroplatinic acid hexahydrate (H_2_PtCl_6_·6H_2_O), 2-aminoterephthalic Acid (BDC-NH_2_), polyvinylpyrrolidone (PVP, Mw=58000), *N,N*-dimethylformamide (DMF), acetic acid, tris(2-carboxyethyl)phosphine hydrochloride (TCEP), *N*-hydroxysuccinimide (NHS), and 1-ethyl-3-(3dimethylaminopropyl) carbodiimide hydrochloride (EDC) were obtained from Aladdin Industrial Co., ltd (Shanghai, China). Tris(2,2’-bipyridine) dichlororuthenium (Ru(bpy)_3_Cl_2_) and tris(4,4'-dicarboxylicacid-2,2'-bipyridyl)ruthenium dichloride (Ru(dcbpy)_3_Cl_2_) were obtained from Macklin Industrial Co., ltd (Shanghai, China). AEC peroxidase substrate kit was purchased from Solaibio life science Co., ltd (Beijing, China). Streptavidin magnetic beads was purchased from MedChemExpress Co., ltd (New Jersey, USA). SYBR Green Premix Pro Taq HS qPCR Kit was obtained from Accurate Biotechnology Co., ltd (Hunan, China). MagaBio plus virus DNA/RNA purification kit was purchased from Bioer technology Co., ltd (Hangzhou, China).

## Apparatus

Transmission electron microscope (TEM) was conducted with a HT7700 microscope (Hitachi, Japan). Scanning electron microscopy (SEM) image was obtained through a SU8010 microscope (Hitachi, Japan). The UV-vis spectra and absorbance were measured by Lambda 365 UV/VIS spectrophotometer (PerkinElmer, USA). The fluorescence spectra and fluorescence values were obtained from on a RF-6000 fluorescence spectrophotometer (Shimadzu, Japan). Zeta potential was determined by Litesizer 500 (Anton Paar, Austria)**.** X-ray diffraction (XRD, D8 ADVANCE, Bruker, Germany) and X-ray photoelectron spectroscopy (XPS, Escalab 250Xi, Thermo Fisher, UK) were used to monitor the crystal structure and elements of Ru@U6-Ru/Pt NPs, respectively. Inductively coupled plasma mass spectrometry (ICP-MS, ICAP RQ, Thermo Fisher, Germany) was used to evaluate the content of Ru@U6-Ru/Pt NPs. The nanoprobes dispensed over the test and control line on the nitrocellulose membrane were sprayed by the HM3035-XYZ 3D film spraying apparatus (Jieyi, Shanghai, China). Optical images of the test strips were acquired by Amersham ImageQuant 800 (Cytiva, Sweden).

## Ethical Statement

The studies involving nucleic acid samples from human subjects were reviewed and approved by the Shenzhen Third People's Hospital (No. 2023-013-02). All subjects provided written informed consent.

## Synthesis of PtNPs

PtNPs were synthesized according to the literature with minor modifications. 20 mg of PVP (Mw=58000) was dissolved in 45 mL of ethanol, and then 5.0 mL of H_2_PtCl_6_ aqueous solution (6.0 mM) was added dropwise. After stirring for about 2 min at room temperature, the solution was refluxed in a 100 mL round flask for 3 h under air to synthesize the PVP-stabilized PtNPs. The concentration of as-synthesized PtNPs was about 0.6 mM and used directly without further treatment.

## Synthesis of AuNPs

The aqueous HAuCl_4_ solution (0.5 mL, 1.0 wt.%) and ultrapure water (50.0 mL) were mixed and boiled. Trisodium citrate solution (0.8 mL, 1.0 wt.%) was rapidly injected into the boiling mixture. After the mixture was stirred for 10 min under boiling, the colloidal solution was AuNPs.

## Synthesis of the capture probe (MB-SA-biotin-DNA)

The synthesis of the capture probe (MB-SA-biotin-DNA) was according to the instructions provided by the manufacturer. Briefly, 100 µL of streptavidin magnetic beads were transferred to a fresh 1.5 mL EP tube, and placed on a magnetic rack for separation, and then the supernatant was discarded. Then, 1 mL of Wash Buffer I (10 mM Tris-HCl, 1 mM EDTA, 1 M NaCl, 0.01% - 0.1% Tween-20, pH 7.5) was added to wash the magnetic beads, and then the beads was separated to discard the supernatant. Biotinylated test DNA (500 μL, final concentration=2 µM) was added into the streptavidin magnetic beads, and then the mixture was incubated on a rotary mixer (room temperature, 30 min) to construct the capture probe (MB-SA-biotin-DNA). The MB-SA-biotin-DNA were then collected, and then washed three times with Wash Buffer I. Finally, MB-SA-biotin-DNA was resuspended in 500 µL of ultrapure water.

# Tables

**Table S1** Sequence of the oligonucleotides used in this study.

| **Name** | **Sequences (5' to 3')** |
| --- | --- |
| MPXV | GTATTGTTGTCTTTACATTTTCCATTGGATGGTGCATG |
| Capture DNA | TGTAAAGACAACGAATACAAAAAAAAAA-SH |
| Test DNA | Biotin-AAAAAAAAAACATGCACCATCCAATGGA |
| Control DNA | Biotin-AAAAAAAAAATCCATTGGATGGTGCATG |
| Camelpox | GTATTCGTTGTCTTTACACTTTCCATTGGATGGTGCATA |
| Cowpox | GTATTCGTTGTCTTTACACTTTCCGTTGGATGGTTCATG |
| Ectromelia | ATAGTCTGTACCGTTACATTTCCCATTAATGGGTGTATA |
| Taterapox | GTATTCGTTGTCTTTACACTTTCCATTGGATGGTGCATA |
| Variola | AATATGCAATCATTACTGTTCCCATGGCTATTACCCACT |
| Forward primer for qPCR | ACGCTAAATTAACGTCTACCGAAACATCGT |
| Reverse primer for qPCR | GGTGGAATTCACTTCGTATAATGGCTTATC |

**Table S2** Apparent steady-state kinetic parameters for Ru@U6-Ru/PtNPs and other PtNPs-modified metal organic frameworks nanozymes.

| **Enzymes or**  **Enzyme mimics** | ***K_m_* (mM)** | | | ***V_max_* (10^-8^ M∙s^-1^)** | | **Ref.** |
| --- | --- | --- | --- | --- | --- | --- |
|  | **TMB** | **H_2_O_2_** | **TMB** | | **H_2_O_2_** |  |
| NH_2_-UiO-66@PtNPs | 0.5 | / | 380 | | / | ^[1]^ |
| Pt-PCN-224 | 3.45 | 0.431 | 226.8 | | 32.4 | ^[2]^ |
| Pt/NH_2_-MIL-101 | 0.12 | 0.48 | 11.5 | | 19.7 | ^[3]^ |
| PtNP@UiO-66-NH_2_ | 0.127 | 36.5 | 1.36 | | 4.15 | ^[4]^ |
| Pt/UiO-66 | 0.20 | 0.18 | 21.12 | | 14.23 | ^[5]^ |
| HRP | 0.43 | 3.70 | 10.00 | | 8.71 | ^[6]^ |
| PtNPs | 0.20 | 11.50 | 3.64 | | 6.11 | This work |
| Ru@U6-Ru/PtNPs | 0.08 | 6.87 | 1.82 | | 5.27 | This work |

**Table S3** Comparison of detection performance of LFA-based methods for MPXV or other nucleic acid targets

| Method | Target | Amplification | LOD | Sensing range | Time  (min) | Ref |
| --- | --- | --- | --- | --- | --- | --- |
| CuO NPs-LFA | HPV16 | × | 1 nM | 5 – 100 nM | 20 | ^[7]^ |
| Carbon nanotube-LFA | miRNA | × | 0.1 nM | 0.1 – 20 nM | 20 | ^[8]^ |
| QDs based with amplification-LFA | HIV | √ | 0.76 pM | 1 pM – 10 nM | 195 | ^[9]^ |
| PCR-immunochromatographic test | HPV | √ | 100 (gene copies) | 0 – 10^4^ (plasmids copy) | >120 | ^[10]^ |
| Electrochemical paper-based peptide nucleic acid biosensor | HPV | × | 2.3 nM | 10 – 200 nM | 45 | ^[11]^ |
| Upconversion nanoparticle‑based LFA | MPXV | × | 1 pM | 0.01 – 50 nM | 8 | ^[12]^ |
| LAMP-LFB | MPXV | √ | 12.5 copies (pseudotyped virus) | 16 copies – 10^3^ copies per reaction | 60 | ^[13]^ |
| AuNPs-LFA | MPXV | × | 6.25×10^4^ pM | 6.25×10^4^ – 5 ×10^5^ pM | 10 | This work |
| General Ru@U6-Ru/Pt NPs based LFA | MPXV | × | 10^3^ pM | 10^3^ – 5 ×10^5^ pM | 7 | This work |
| Colorimetric Ru@U6-Ru/Pt NPs based LFA | MPXV | × | 0.1 pM | 0.1 – 5 ×10^5^ pM | 8 | This work |

**Table S4** Comparison of detection performance with existing quantitative detection assay for MPXV

| Method | Amplification | LOD  (copies∙µL^–1^) | Sensing range (copies∙µL^–1^) | Nucleic acid extraction | Time  (min) | Ref |
| --- | --- | --- | --- | --- | --- | --- |
| CRISPR-SPR-FT sensor | × | 59.5 | 10^2^ –10^6^ | √ | 90 | ^[14]^ |
| Single-step RPA-CRISPR/Cas12a | √ | 15 | 6 – 30  and 6 – 6 ×10^5^ | √ | 35 | ^[15]^ |
| LAMP | √ | 28.7 | 1 – 10^3^ | √ | 30 | ^[16]^ |
| DNAzyme-based  chemiluminescence  CRISPR/Cas12a | √ | 5.2 | 6 – 180 | √ | > 60 | ^[17]^ |
| RPA, CRISPR/Cas12a,  nanopore | √ | 16 | 1 – 10^5^ | √ | 55 | ^[18]^ |
| Single-step RPA-CRISPR/Cas12a | √ | 10.6 | 6 – 6.9 × 10^5^ | × | 35 | ^[19]^ |
| Ru@U6-Ru/Pt NPs based ECL platform | × | 6 | 60 – 6 × 10^9^ | × | < 15 | This work |

# Figures

**
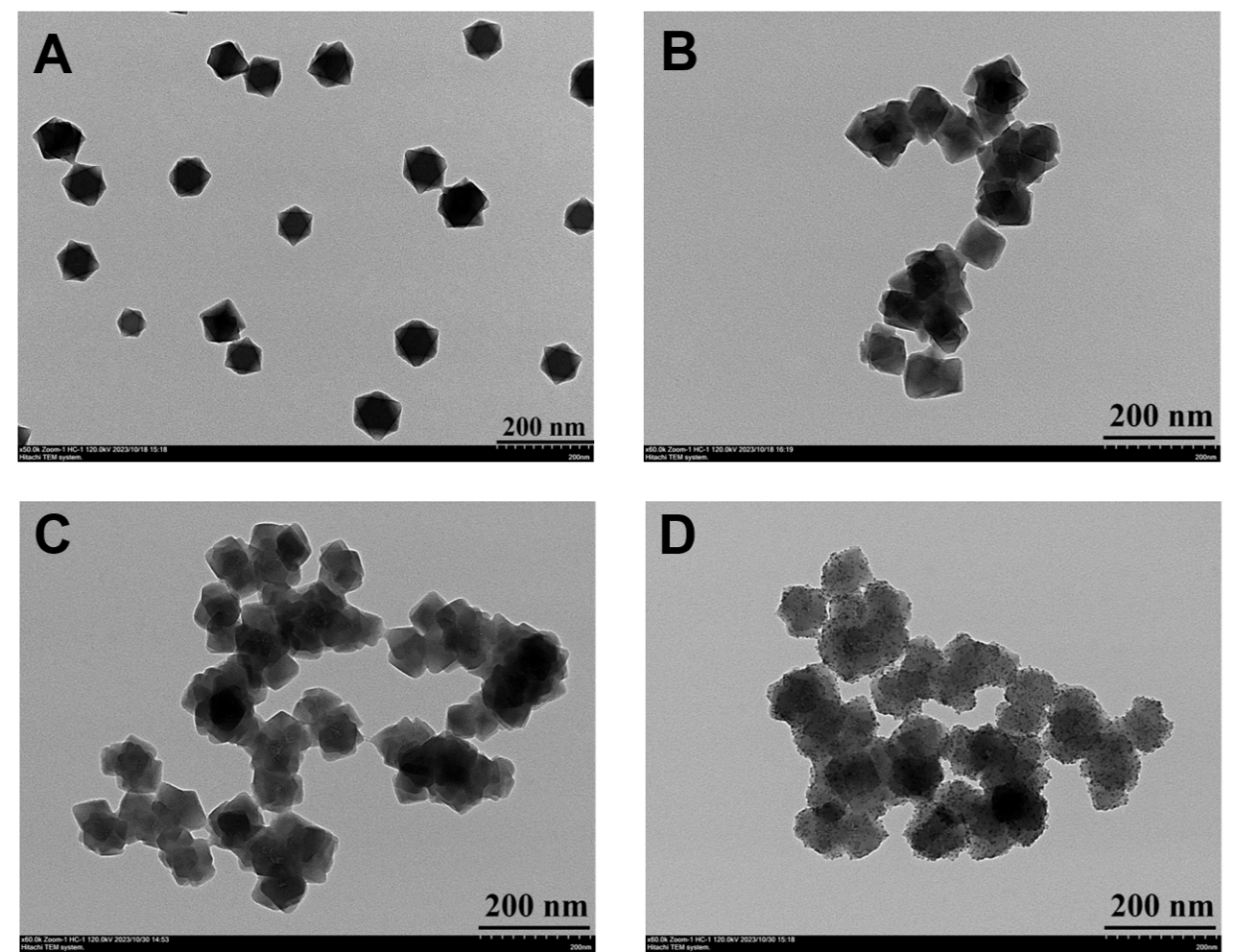
**

**Figure S1.** TEM images of (A) U6-NH_2_ NPs, (B) Ru@U6-NH_2_ NPs, (C) Ru@U6-Ru NPs, and (D) Ru@U6-Ru/Pt NPs.

**
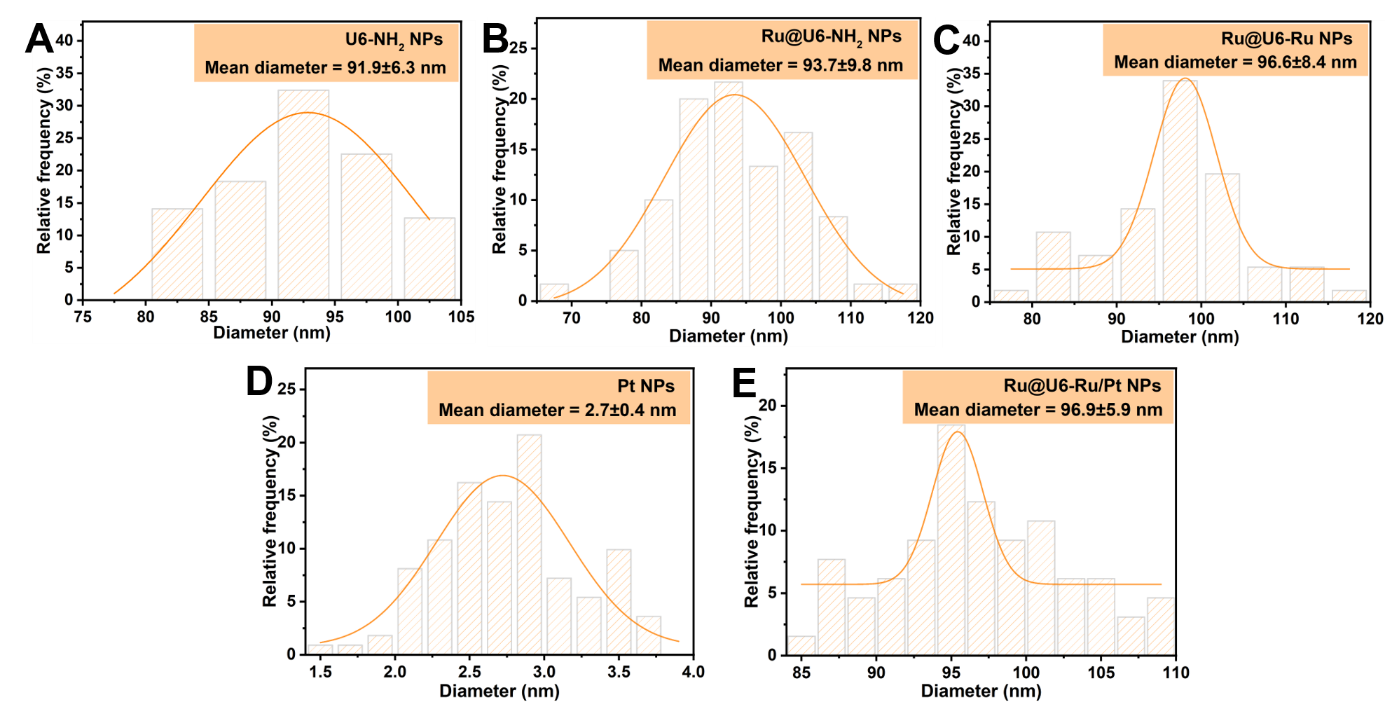
**

**Figure S2.** The corresponding histograms of diameters for (A) U6-NH_2_ NPs, (B) Ru@U6-NH_2_ NPs, (C) Ru@U6-Ru NPs, (D) PtNPs and (E) Ru@U6-Ru/Pt NPs.


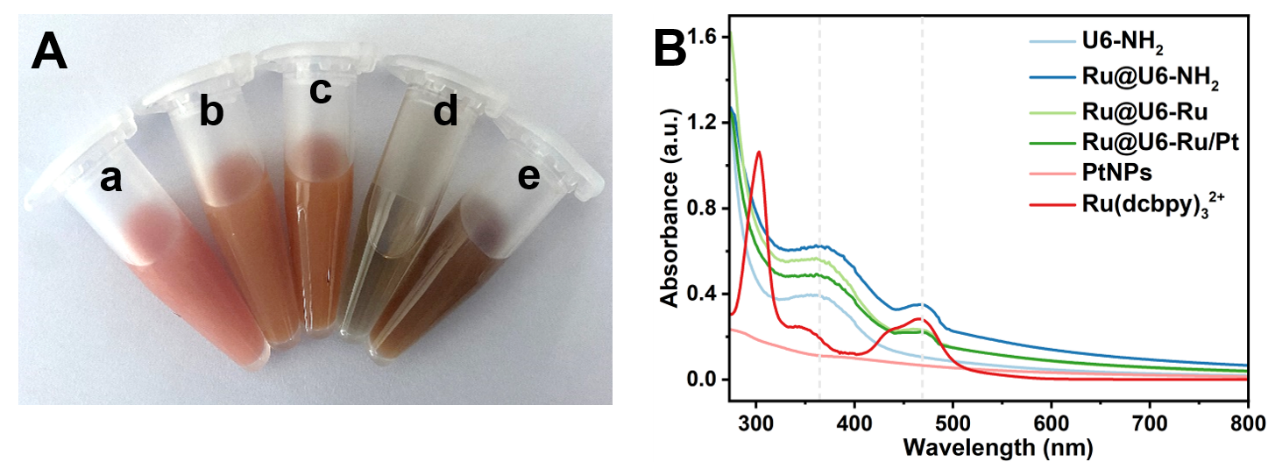


**Figure S3.** (A) Photographs of (a) U6-NH_2_ NPs, (b) Ru@U6-NH_2_ NPs, (c) Ru@U6-Ru NPs, (d) PtNPs, and (e) Ru@U6-Ru/Pt NPs.

**
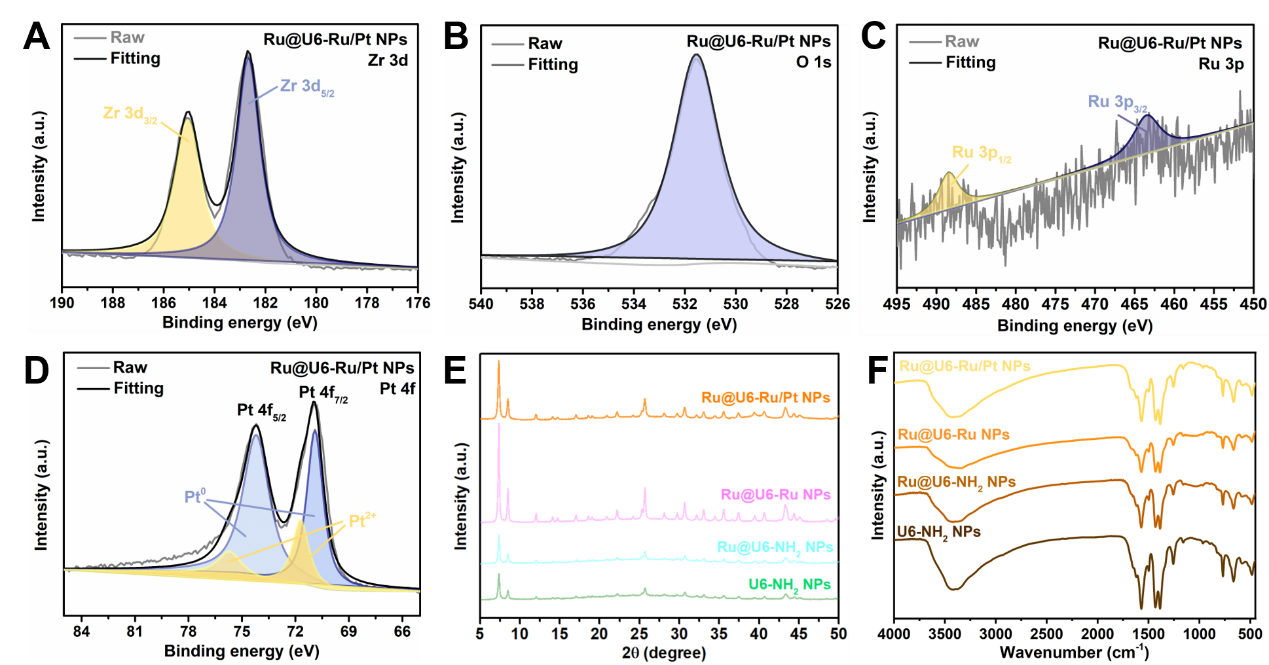
**

**Figure S4.** High-resolution (A) Ru 3p, (B) Zr 3d, (C) Pt 4f and; and (D) O 1S XPS spectra of Ru@U6-Ru/Pt NPs. (E) XRD pattern of U6-NH_2_ NPs, Ru@U6-NH_2_ NPs, Ru@U6-Ru NPs, and Ru@U6-Ru/Pt NPs. (F) FT-IR pattern of U6-NH_2_ NPs, Ru@U6-NH_2_ NPs, Ru@U6-Ru NPs, and Ru@U6-Ru/Pt NPs.


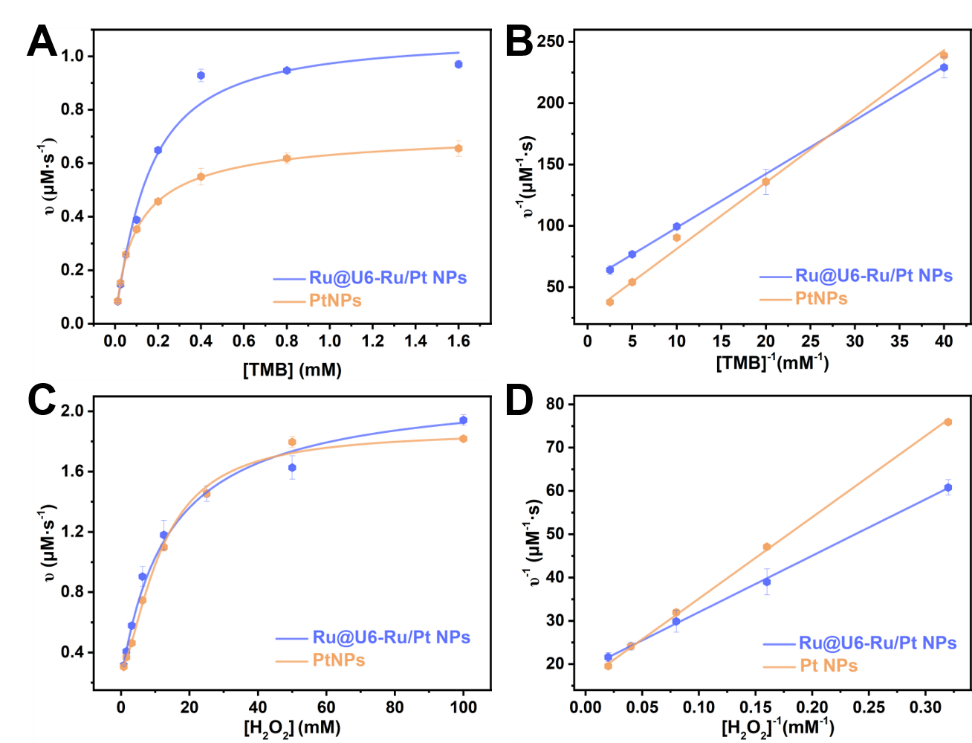


**Figure S5.** Steady-state kinetic assay of Ru@U6-Ru/Pt NPs based on POD-like activity. (A) TMB concentration and (C) H_2_O_2_ concentration dependence of initial reaction velocity (*v*), double reciprocal plots between reaction velocity and (B)TMB concentration and (D) H_2_O_2_ concentration. Error bars denote the standard deviation (*n* = 3).


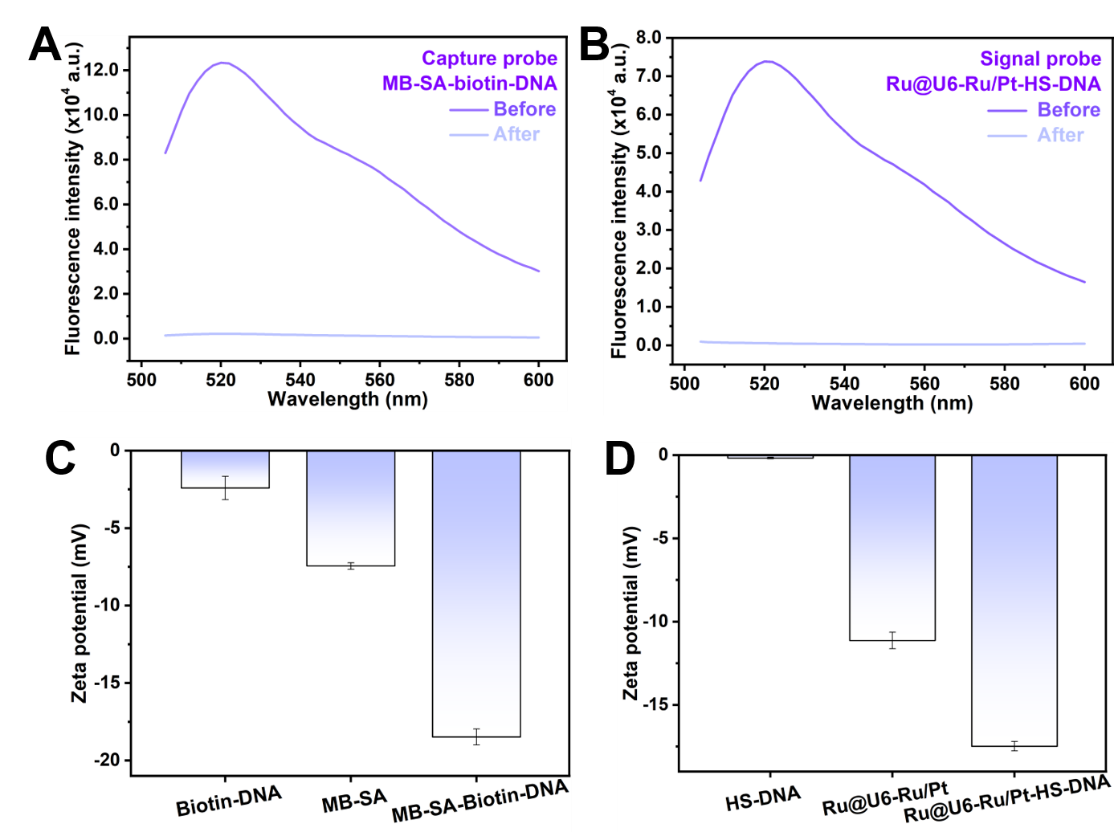


**Figure S6.** Characterization of capture probes and signal probes. Fluorescence spectra of supernatant before and after modification to form (A) capture probe MB-SA-biotin-DNA and (B) signal probe Ru@U6-Ru/Pt-HS-DNA. (C) Zeta potential of Biotin-DNA, MB-SA, and MB-SA-biotin-DNA. (D) Zeta potential of HS-DNA, Ru@U6-Ru/Pt, and Ru@U6-Ru/Pt-HS-DNA. Error bars denote the standard deviation (*n* = 3).

**
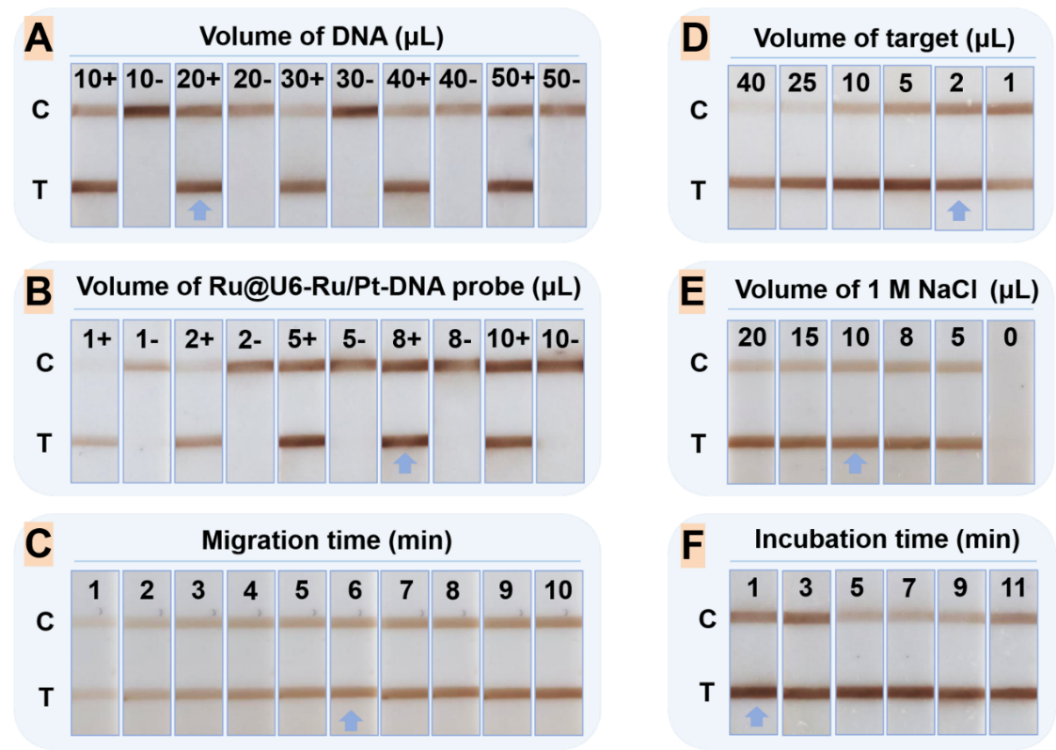
**

**Figure S7.** Optimization photos of crucial experimental parameters for Ru@U6-Ru/Pt-based LFA. (A) Volume of DNA. (B) Volume of Ru@U6-Ru/Pt-DNA probe. (C) Migration time. (D) Volume of target. (E) Volume of NaCl. (F) Incubation time.

**
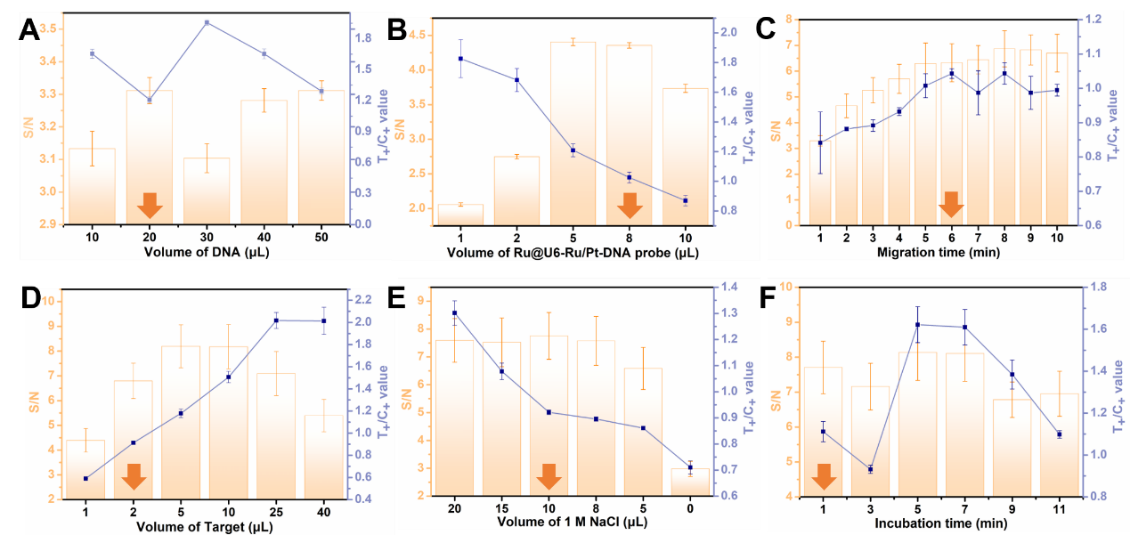
**

**Figure S8.** Optimization results of crucial experimental parameters for Ru@U6-Ru/Pt-based LFA. (A) Volume of DNA. (B) Volume of Ru@U6-Ru/Pt-DNA probe. (C) Migration time. (D) Volume of target. (E) Volume of NaCl. (F) Incubation time. Error bars denote the standard deviation (*n* = 3).


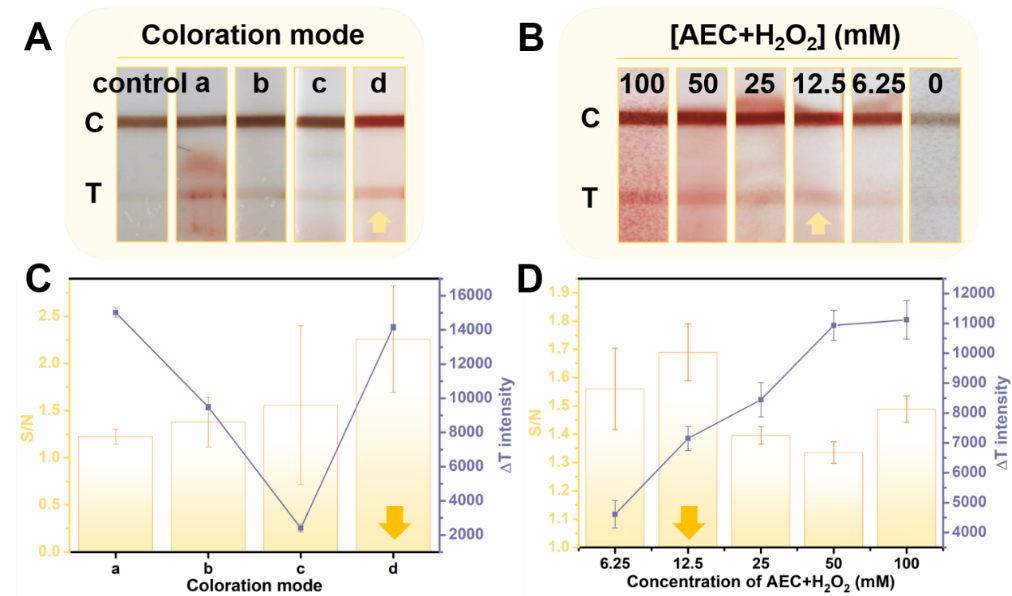


**Figure S9.** (A, C) Effect of different loading modes of AEC and H_2_O_2_ mix solution. (a) loading on the test line; (b) loading on sample pad; (c) partial immersion in the tube; (d). (B, D) Effect of different concentration of AEC and H_2_O_2_ mix solution. Error bars denote the standard deviation (*n* = 3).


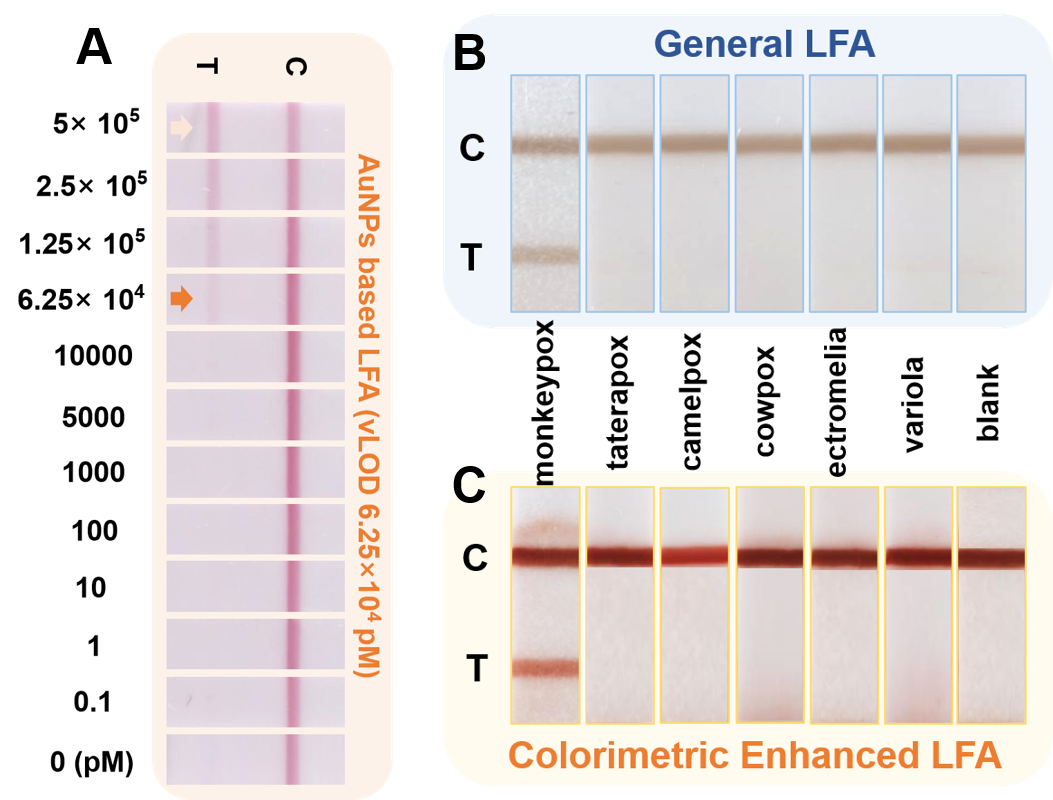


**Figure S10.** (A) Photographs of the AuNPs based LFA in response to different concentrations of MPXV. (B) Selectivity of general Ru@U6-Ru/Pt-based LFA and (C) enhanced Ru@U6-Ru/Pt-based LFA.


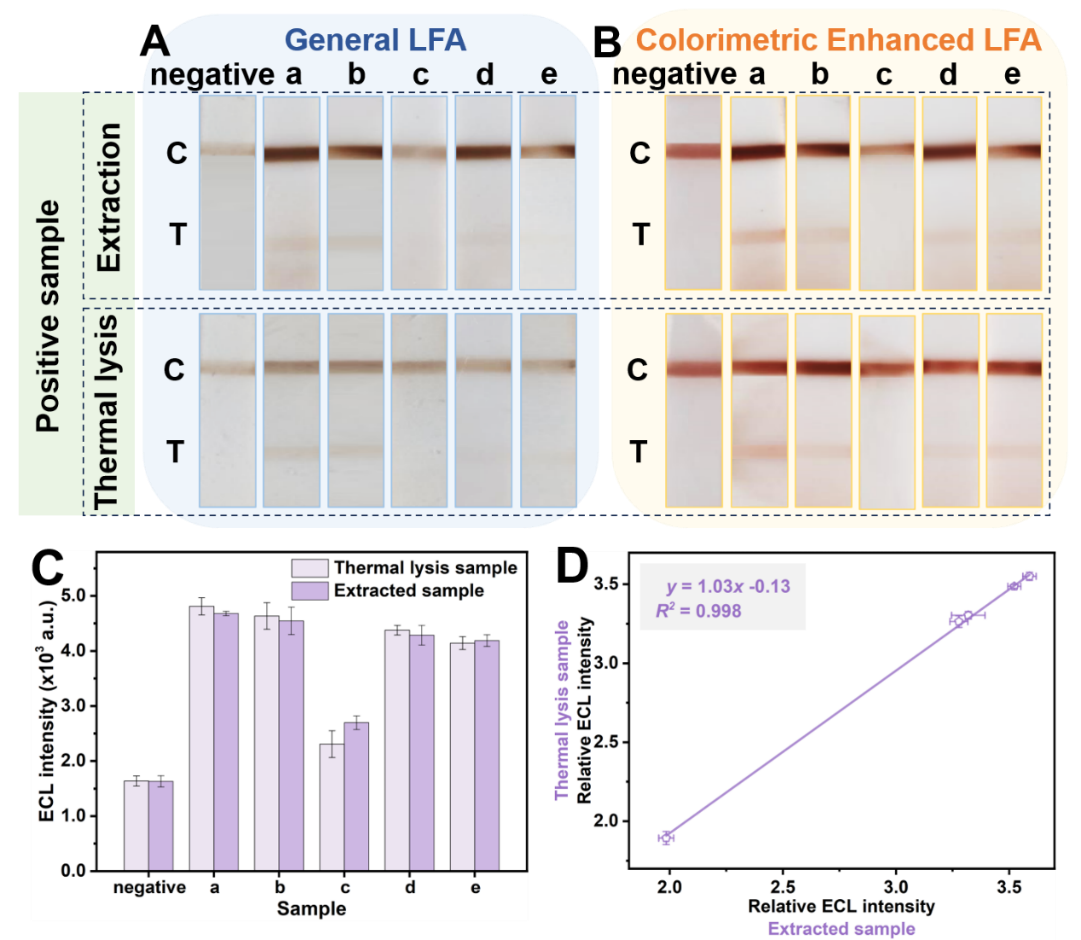


**Figure S11.** The photographs of positive and negative thermal lysis and extracted samples detected by (A) general Ru@U6-Ru/Pt NPs based LFA and (B) colorimetric enhanced Ru@U6-Ru/Pt NPs based LFA. (C) The ECL responses of positive and negative thermal lysis and extracted samples detected by the Ru@U6-Ru/Pt NPs based ECL biosensor. (D) Correlation between thermal lysis samples and extracted samples detected by the Ru@U6-Ru/Pt NPs based ECL biosensor. Error bars denote the standard deviation (*n* = 3).


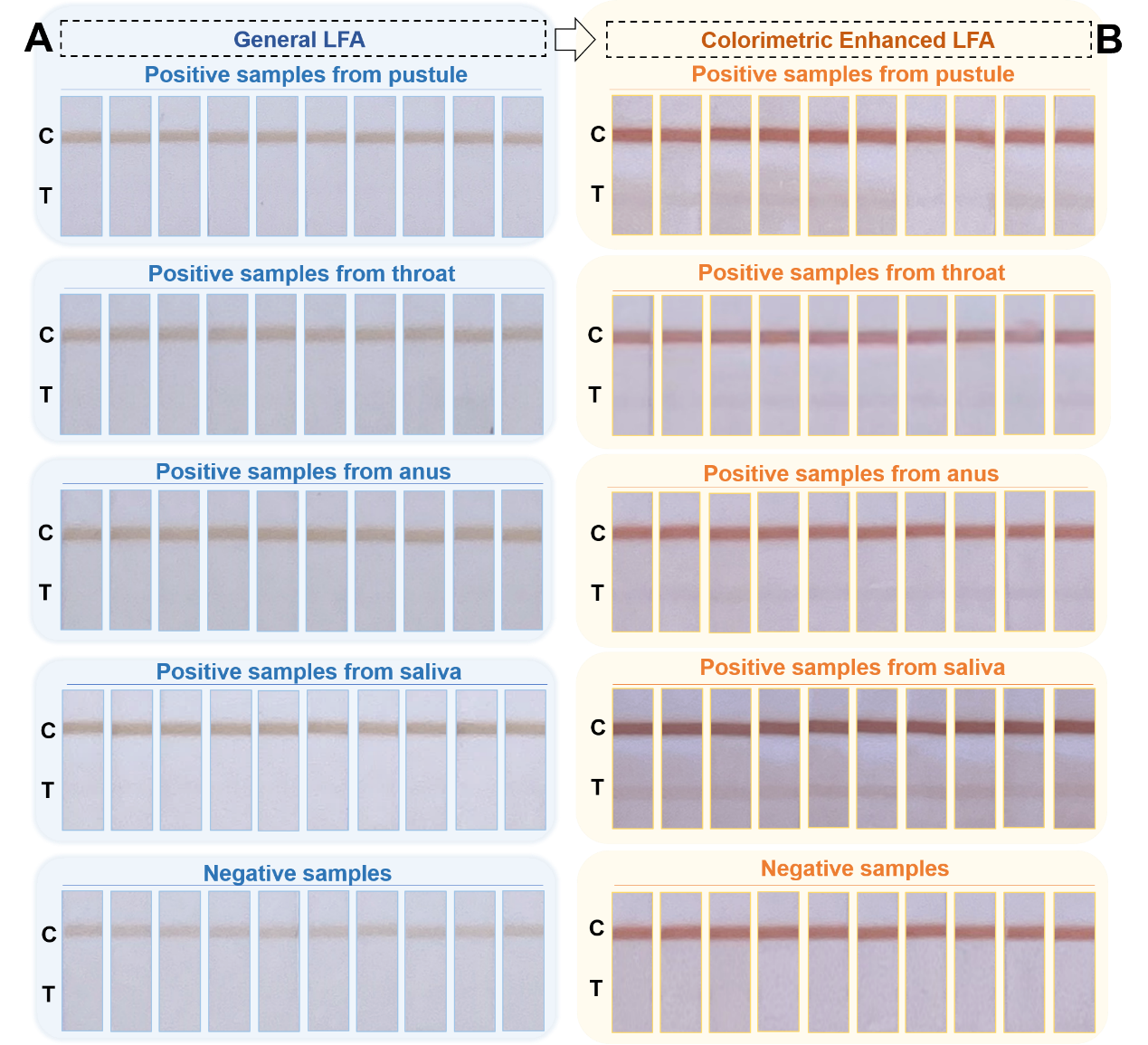


**Figure S12.** Results of MPXV-positive samples collected from pustule, throat, anus, and saliva and MPXV-positive samples tested by (A) general Ru@U6-Ru/Pt NPs based LFA and (B) colorimetric enhanced Ru@U6-Ru/Pt NPs based LFA.


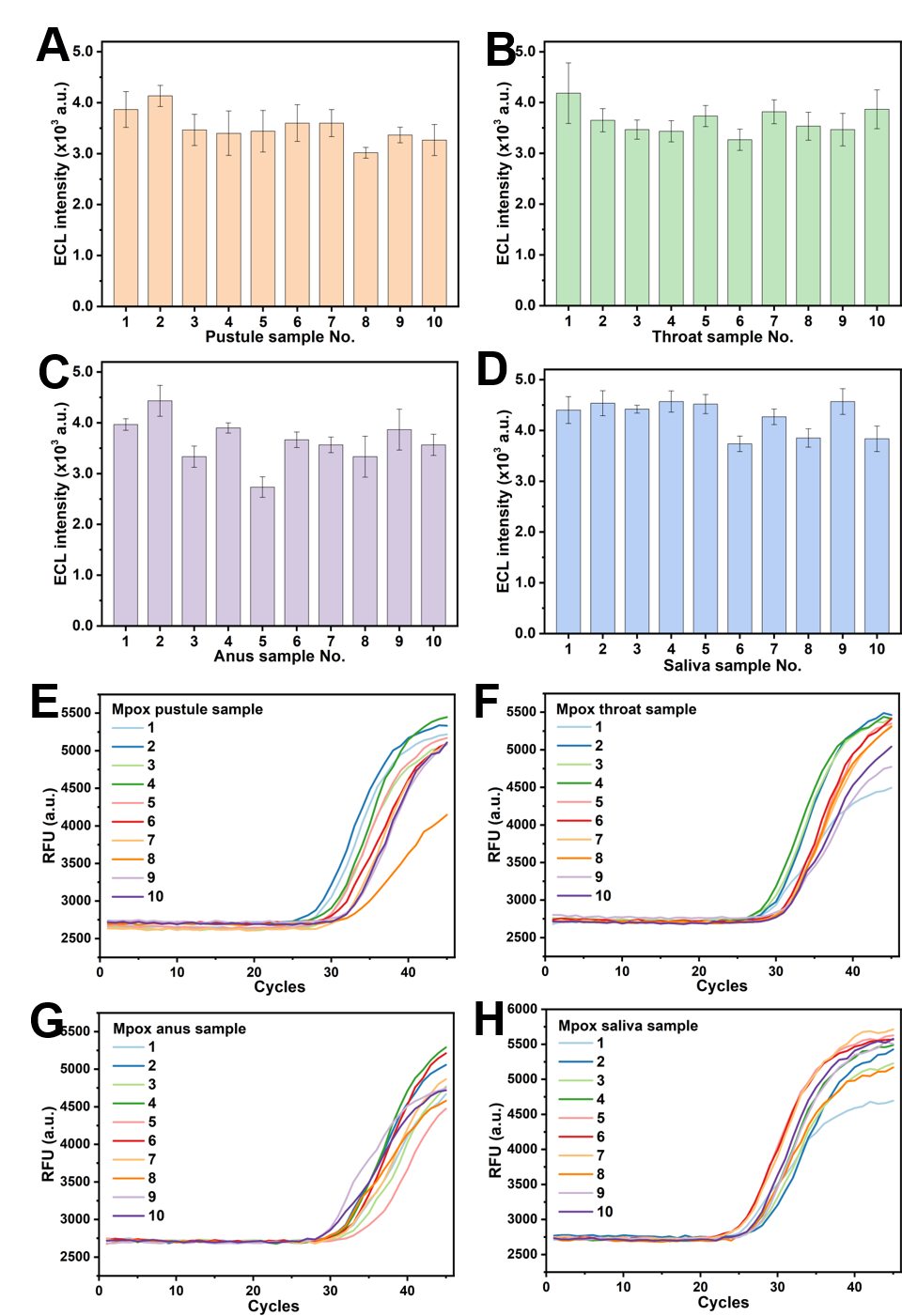


**Figure S13.** ECL responses of MPXV-positive samples collected from (A) pustule, (B) throat, (C) anus, and (D) saliva and detected by the Ru@U6-Ru/Pt NPs based ECL biosensor. qPCR results for MPXV-positive samples collected from (E) pustule, (F) throat, (G) anus, and (H) saliva. Error bars denote the standard deviation (*n*=3).

# References

[1] H. Li, J. Yang, X. Hu, R. Han, S. Wang, M. Pan, *Chemical Engineering Journal* **2023**, *473*, 145401.

[2] G. Xing, Y. Shang, J. Ai, H. Lin, Z. Wu, Q. Zhang, J.-M. Lin, Q. Pu, L. Lin, *Anal. Chem.* **2023**, *95*, 13391-13399.

[3]

[4] H. Li, H. Liu, J. Zhang, Y. Cheng, C. Zhang, X. Fei, Y. Xian, *ACS Applied Materials & Interfaces* **2017**, *9*, 40716-40725.

[5] H. Wang, J. Zhao, C. Liu, Y. Tong, W. He, *ACS Omega* **2021**, *6*, 4807-4815.

[6] J. Li, G. Zhang, L. Wang, A. Shen, J. Hu, *Talanta* **2015**, *140*, 204-211.

[7] Z. Yang, C. Yi, S. Lv, Y. Sheng, W. Wen, X. Zhang, S. Wang, *Sensors and Actuators B: Chemical* **2019**, *285*, 326-332.

[8] W. Qiu, H. Xu, S. Takalkar, A. S. Gurung, B. Liu, Y. Zheng, Z. Guo, M. Baloda, K. Baryeh, G. Liu, *Biosensors and Bioelectronics* **2015**, *64*, 367-372.

[9] X. Deng, C. Wang, Y. Gao, J. Li, W. Wen, X. Zhang, S. Wang, *Biosensors and Bioelectronics* **2018**, *105*, 211-217.

[10] Y.-B. Kuo, Y.-S. Li, E.-C. Chan, *J. Virol. Methods* **2015**, *212*, 8-11.

[11] P. Teengam, W. Siangproh, A. Tuantranont, C. S. Henry, T. Vilaivan, O. Chailapakul, *Analytica Chimica Acta* **2017**, *952*, 32-40.

[12] B. R. Jin, C. Ma, C. Y. Zhang, H. L. Yin, G. X. Zhao, J. Hu, Z. D. Li, *MICROCHIMICA ACTA* **2024**, *191*,

[13] F. Xiao, J. Fu, X. Huang, N. Jia, C. Sun, Z. Xu, H. Huang, J. Zhou, Y. Wang, *Talanta* **2024**, *269*, 125502.

[14] Y. Chen, Z. Chen, T. Li, M. Qiu, J. Zhang, Y. Wang, W. Yuan, A. H.-P. Ho, O. Al-Hartomy, S. Wageh, A. G. Al-Sehemi, X. Shi, J. Li, Z. Xie, L. Xuejin, H. Zhang, *ACS Nano* **2023**, *17*, 12903-12914.

[15] Q. Chen, I. Gul, C. Liu, Z. Lei, X. Li, M. A. Raheem, Q. He, Z. Haihui, E. Leeansyah, C. Y. Zhang, V. Pandey, K. Du, P. Qin, *J. Med. Virol.* **2023**, *95*, e28385.

[16] Y. Zeng, Y. Zhao, X. Ren, X. Zhou, C. Zhang, Z. Wan, Y. Q. Kuang, *J. Infect.* **2023**, *86*, e114-e116.

[17] H. Chen, Y. Feng, F. Liu, C. Tan, N. Xu, Y. Jiang, Y. Tan, *Biosensors and Bioelectronics* **2024**, *247*, 115929.

[18] M. A. Ahamed, M. A. U. Khalid, M. Dong, A. J. Politza, Z. Zhang, A. Kshirsagar, T. Liu, W. Guan, *Biosensors and Bioelectronics* **2024**, *246*, 115866.

[19] J. Wei, W. Wang, Q. Yu, M. Zhang, F. Xue, B. Fan, T. Zhang, Y. Gao, J. Li, X. Meng, B. Pang, *Sensors and Actuators B: Chemical* **2023**, *390*, 133950.
